# Direct entry of micro(nano)plastics into human blood circulatory system by intravenous infusion

**DOI:** 10.1016/j.isci.2023.108454

**Published:** 2023-11-14

**Authors:** Penghui Li, Qingcun Li, Yujian Lai, Shuping Yang, Sujuan Yu, Rui Liu, Guibin Jiang, Jingfu Liu

**Affiliations:** 1 School of Environment, Hangzhou Institute for Advanced Study, University of Chinese Academy of Sciences, Hangzhou 310024, China; 2 State Key Laboratory of Environmental Chemistry and Ecotoxicology, Research Center for Eco-Environmental Sciences, Chinese Academy of Sciences, Beijing 100085, China; 3 School of Environmental Science and Engineering, Southern University of Science and Technology, Shenzhen 518055, China; 4 Hubei Key Laboratory of Environmental and Health Effects of Persistent Toxic Substances, Institute of Environment and Health, Jianghan University, Wuhan 430056, China

**Keywords:** Health sciences, Environmental science, Pollution

## Abstract

Understanding the pathways of human exposure to micro(nano)plastics (MNPs) is crucial for assessing their health impacts. Intravenous infusion can induce MNPs direct entry into the human blood, posing serious risks on human health, but remains unclear. Herein, we developed comprehensive analytical methods to detect polyvinyl chloride (PVC) MNPs down to 20 nm, and found about 0.52 μg equal to 10^5^–10^11^ particles of PVC-MNPs released from intravenous infusion products (IVIPs) during each intravenous infusion of 250 mL injection. The released amounts of MNPs from IVIPs were dependent on the plastic materials, and the injection volume and composition. These findings indicated that the released MNPs should be directly introduced into the human blood circulatory system, causing serious impacts on human health. Our study reveals a previously ignored but important pathway of human exposure to MNPs, and calls for further research on the potential risks of these MNPs on human health.

## Highlights

• A comprehensive method for detecting micro(nano)plastics (MNPs) was developed
• Intravenous infusion products can release MNPs during intravenous infusion
• MNPs can directly enter the human blood circulatory system via intravenous infusion
• Mass and number of MNPs entering human via intravenous infusion were assessed

## Graphical abstract

**Figure:**
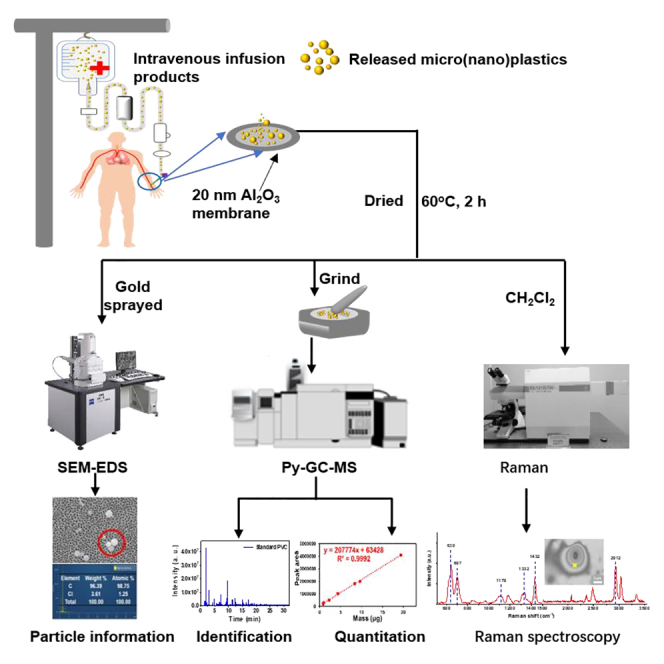

## Introduction

Increasing attention has been paid to micro(nano)plastics (MNPs), including microplastics (MPs, <5 mm) and nanoplastics (NPs, <1 μm),^1^^,^^2^^,^^3^ due to their worldwide occurrence and potential risks to surroundings, living organisms, (eco)systems, and even human health.^4^^,^^5^^,^^6^^,^^7^^,^^8^ Studies have confirmed the presence of MPs in the human body, such as lung tissue, thrombi, placenta, blood, and breast milk.^9^^,^^10^^,^^11^^,^^12^^,^^13^ Meanwhile, a growing number of studies revealed that NPs can penetrate plants and other organisms, leading to neurotoxicity, oxidative damage, obesity, and other negative effects because of their smaller sizes.^14^^,^^15^^,^^16^^,^^17^ Despite this belated recent surge in research on MNPs and human health, their impacts on human health at large are still poorly known,^18^^,^^19^ which is greatly impeded by the lack of knowledge on human exposure to MNPs.^7^^,^^20^ Understanding the exposure routes of MNPs to the human body is thus crucial.

Current studies mainly focus on inhalation and ingestion routes, which are considered as the dominant pathways for MNPs to enter the human body.^7^ For example, MPs have been observed in drinking water, salts, seafood, and other foods, and MNPs were also demonstrated to release from silicone-rubber baby teats, feeding bottles, cutting boards, plastic cups, take-out food containers, and other plastic containers during their usage.^21^^,^^22^^,^^23^^,^^24^^,^^25^^,^^26^ All these MNPs can enter the human body through ingestion during our daily diet. On the other hand, MPs can also be inhaled through regular breath due to their widespread distributions in atmosphere and indoor air.^27^^,^^28^ Although it was estimated that an adult takes up more than 30,0000 MPs/year through inhalation and ingestion,^29^ these MPs could be excreted out through human sputum and feces, resulting in reduced risks to the human body.^30^^,^^31^ In addition, studies have demonstrated that MNPs can be translocated to other organs in mice,^32^^,^^33^ but it is quite plausible that this is also the case for humans. However, remarkable MPs were identified in human blood very recently, and their source remains unclear.^12^ Therefore, there may exist other pathways for MNPs to directly entry into the human blood, such as intravenous injection, and related research is urgently needed.

Intravenous therapy including parenteral nutrition as a special “food” is commonly administrated to various patients. The number of patients receiving total parenteral nutrition was estimated as 33,000 per year with steady increase over the past decade in the United States.^34^^,^^35^ Meanwhile, it is also reported that the average annual per capita intravenous infusion is ∼2.5–3.5 bottles/bags globally and ∼7.5 bottles/bags in China.^36^ Previous studies have demonstrated that intravenous infusion products (IVIPs) could release microparticles using the optical microscope based on standard methods recommended by the International Organization for Standardization (ISO), which may cause thrombosis, organ dysfunction, and other negative effects on human.^37^^,^^38^^,^^39^ However, the standard methods can only detect large particles (>25 μm),^40^ and are not applicable for counting the number and identifying the components for the smaller particles, especially nanoscale particles that are potentially more toxic. Given most IVIPs are currently made of plastics,^41^ we speculate that MNPs could be released from these plastic components and directly enter human blood circulatory system during intravenous infusion. It is believed that once entering human blood circulatory system, the MNPs can gradually accumulate and eventually be transported to the brain, heart, and other important organs, which may be related to cerebral thrombosis, cardiovascular disease, and other severe diseases threatening human health.^25^ However, this direct exposure route of MNPs by intravenous infusion has not been verified up to now, mainly due to the lack of analytical methods for identifying and quantifying the released MNPs.

With analytical technique for MNPs moving forward, identification and quantitation of these MNPs can be achieved. For example, pyrolysis-gas chromatography-mass spectrometry (Py-GC-MS) is a powerful technique to identify and quantify MNPs in complex environmental matrixes^41^^,^^42^^,^^43^ with low sample consumption and high mass sensitivity,^44^^,^^45^ which can also be used to identify and quantify MNPs released from IVIPs during intravenous infusion. Meanwhile, scanning electron microscopy equipped with energy-dispersive X-ray spectroscopy (SEM-EDS) can provide the size, morphology, and element distributions of the released MNPs. Additionally, direct spectral evidence of the released MNPs can be also obtained by modified Raman spectroscopy. Thus, these effective tools can provide multiple parameters of MNPs released from IVIPs, which makes it possible to comprehensively assess human exposure to MNPs via intravenous infusion.

Polyvinyl chloride (PVC) IVIPs, mainly used in injection bags, injection tubes, blood bags etc., are the most commonly used IVIPs, which account for 26% of the total IVIPs in 2021, equal to approximately 123,400 tons.^46^ What’s worse, studies have indicated that PVC exhibits potential toxic effects on living creatures.^47^^,^^48^ Herein, we selected PVC-IVIPs, including infusion bags, tubes, and sets,^49^ to reveal direct entry of MNPs into the human blood via intravenous infusion. This study aims to (i) develop comprehensive analytical methods suitable for the identification and quantification of PVC-MNPs down to 20 nm, (ii) analyze PVC-MNPs released from PVC-IVIPs, (iii) comprehensively assess human exposure to PVC-MNPs via intravenous infusion, and (iv) explore influence factors influencing the release of PVC-MNPs from PVC-IVIPs. Our study reveals that intravenous infusion is a new but important pathway of human exposure to MNPs, in which MNPs directly enter the human blood circulatory system, posing a serious potential hazard to human health that calls for further assessment.

## Results and discussion

### Characterization of standard PVC and pristine PVC-IVIPs

We characterized the standard PVC particles and pristine Al_2_O_3_ filtering membranes by SEM-EDS. SEM characterization showed that the standard PVC particles were spherical with sizes ranged from 30 to 1650 nm with an average diameter of 480 nm (Figure S1A). The main elemental compositions of these particles were C and Cl (Figure S1B). The SEM images of Al_2_O_3_ membranes indicated that the membrane pore showed a conical structure with a diameter of 20 nm on one side and 200 nm on the other side (Figure S2). This conical structure enhanced the filtration rate but had no influence on the particle cutoff size (20 nm), and was used to collect MNPs in previous studies.^50^^,^^51^ Thus, the Al_2_O_3_ membranes were used to collect ≥20 nm particles in this work.

To verify the applicability of standard PVC as external standards for determining MNPs released from PVC-IVIPs, these two materials were compared by Py-GC-MS and Raman spectroscopy characterization. Both standard PVC and pristine PVC-IVIPs showed characteristic absorption peaks at 639 and 697 cm^−1^ corresponding to the C−Cl stretching vibration in PVC polymer, as well as absorption peaks at 2917, 1432, and 1178 cm^−1^ assigned to the C−H stretching vibration, C−H bending vibration, and C−H rocking vibration, respectively (Figure S3). On the other hand, the main pyrolysis products of standard PVC are nine aromatic compounds, including benzene, methylbenzene, styrene, indene, methyl indene, naphthalene, methylnaphthalene, acenaphthene, and anthracene (Figures S4 and S5; Table S2), which well matched with that of pristine PVC-IVIPs in both the mass spectra and peak times (Figures S4 and S6). These results also demonstrated that standard PVC and pristine PVC-IVIPs shared similarities in their main components. However, pyrolysis products of polypropylene (PP) infusion bottle and non-PVC multilayer co-extrusion film (NPVC) infusion bag were mainly aliphatic hydrocarbons instead of aromatic compounds (Figures S4, S7, and S8), indicating that these two infusion products did not interfere the determination of PVC-MNPs released from PVC-IVIPs (details in Note S1). In addition, we also analyzed the relationship between the main pyrolysis products and mass of PVC using standard PVC (Figure S9), and found that the percentage of benzene (m/z 78) accounts for about 70% of the pyrolysis products in the studied mass range of 0.01–9.58 μg. This indicates that benzene was the most abundant and sensitive pyrolysis product of PVC, which facilitates to determine the low concentration of PVC-MNPs. Thus, benzene is the optimum indicator compound for quantification of PVC-MNPs released from PVC-IVIPs (details in Note S2). Therefore, standard PVC can be used to establish analytical methods for the identification and quantitation of released PVC-MNPs from PVC-IVIPs.

### Development and validation of analytical methods for PVC-MNPs

**Figure 1:**
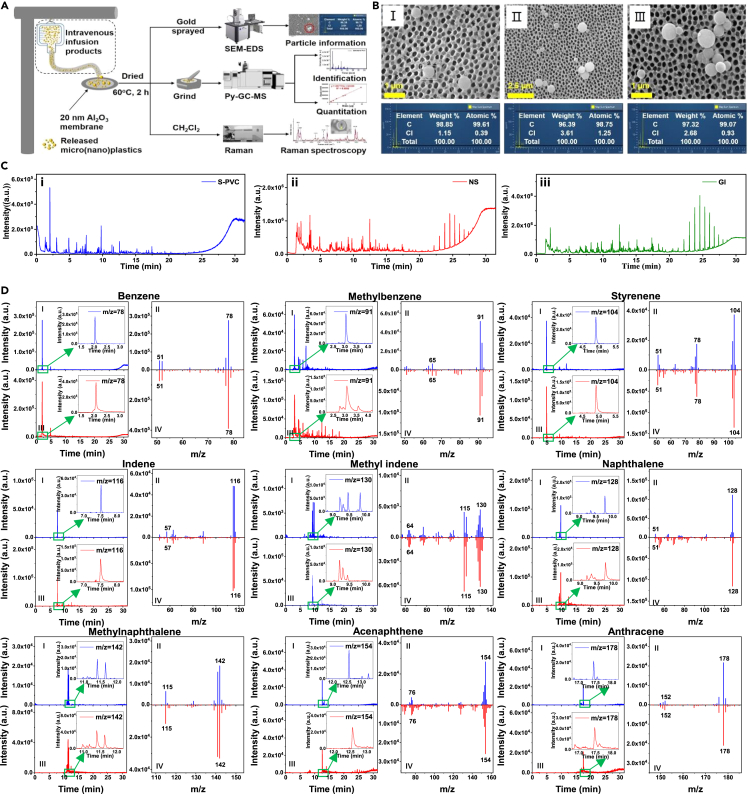
Proposed methods for the analysis of PVC-MNPs (A) Schematic diagram of the main experimental procedure. (B) Typical SEM-EDS images of spiked PVC collected on Al_2_O_3_ membranes, Ⅰ, Ⅱ, and Ⅲ represent the PVC (5 μg) collected by dropping 0.01 mL ethanol spiked with PVC, filtering 500 mL of 0.9% sodium chloride injection (normal saline, NS) spiked with PVC and packed in polypropylene (PP) bottles, and filtering 500 mL of 5% glucose injection (GI) spiked with PVC and packed in PP bottle, respectively. (C) Total ion chromatogram (TIC) of spiked standard PVC, the PVC (5 μg) in i, ii, and iii were collected in the same way as that in Ⅰ, Ⅱ, and Ⅲ of b, respectively. (D) Selected indicator-ion chromatograms (SIC) and mass spectrum (MS) of the nine indicator ions of standard PVC (blue) and spiked PVC added into NS (red). In the SIC and MS of each corresponding indicator ion, Ⅰ and Ⅱ represent SIC of selected indicator ion, Ⅲ and Ⅳ represent MS of selected indicator ion. Each arrow indicates a magnified view of the area inside the green square.

To measure the released MNPs from PVC-IVIPs, injections in PVC-IVIPs were directly filtrated through the 20 nm pore-sized Al_2_O_3_ membranes via simulating intravenous infusion, and the collected substances together with the membranes were analyzed by SEM-EDS and Py-GC-MS (Figure 1A). SEM-EDS analysis showed that the standard PVC particles (5 μg) spiked in 500 mL of 0.9% sodium chloride injection (normal saline, NS) and 5% glucose injection (GI) were both detected on Al_2_O_3_ membranes (Figure S10A), with the main elemental compositions of C and Cl (Figure 1B). However, no particles were observed on Al_2_O_3_ membrane after direct filtration of NS and GI without spiking of PVC (Figure S10B). These results demonstrated that the spiked PVC can be efficiently collected via the proposed method. In addition, all the nine pyrolysis products of PVC were detected for PVC particles spiked in NS and GI, respectively (Figure 1C). Furthermore, the pyrolysis products of PVC-IVIPs (Figures S4 and S6) exhibited excellent consistency with that of standard PVC (Figure 1C) directly measured by Py-GC-MS in both of the mass spectra and peak times. A few pyrolysis products of PVC (such as methylbenzene, methylnaphthalene) were observed in procedure blank without spiking of standard PVC (Figure S11), which probably derived from pyrolysis of some impurities introduced in the production of injections. Even so, this yet did not lead to false positive results as we confirmed the presence of PVC-MNPs only on condition of detecting all of the nine pyrolysis products of PVC (details in Note S3). These results indicated the absence of PVC in the procedure blanks, and the injections scarcely influenced the identification of released PVC-MNPs. Therefore, the spiked PVC can be identified by this method. Meanwhile, the recovery of spiked PVC was also calculated using corrected data (Equation 1 in STAR Methods) according to calibration curves prepared using standard PVC at known quantities based on Py-GC-MS measurements (Figure S12). Results showed that the limit of detection and limit of quantification were 0.0004 and 0.0012 μg, respectively (Table S3), and the recoveries of PVC in NS and GI at different spiking levels ranged from (80.23 ± 12.03)% to (98.37 ± 10.76)% (Table S4), suggesting satisfactory performance of the developed method for the quantitation of actual PVC-MNPs released from PVC-IVIPs. Over all, these previous results demonstrated that the developed method could efficiently collect, identify, and quantify PVC at low mass levels, and thus can be adapted to the analysis of PVC-MNPs released from PVC-IVIPs.

### Identification of MNPs released from PVC-IVIPs

**Figure 2:**
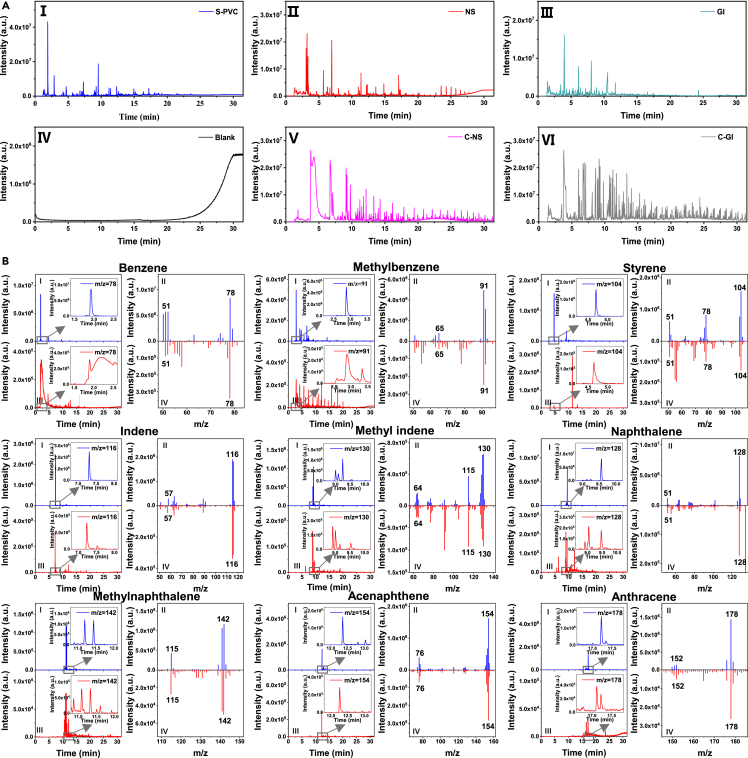
Identification of released PVC-MNPs from PVC-IVIPs based on Py-GC-MS determination (A) Total ion chromatogram (TIC) of standard PVC-MNPs (Ⅰ), filtering 3000 mL of 0.9% sodium chloride injection (normal saline, NS) packed in polypropylene (PP) bottles (Ⅱ), filtering 3000 mL of 5% glucose injection (GI) packed in PP bottles (Ⅲ), air blank (Ⅳ), procedure blank of NS (Ⅴ), procedure blank of GI (Ⅵ). (B) Selected indicator-ion chromatogram (SIC) and mass spectrum (MS) of corresponding indicator ion of standard PVC (blue) and released PVC by the NS injection (red). In the SIC and MS of each corresponding indicator ion, Ⅰ and Ⅱ represent SIC of selected indicator ion, Ⅲ and Ⅳ represent MS of selected indicator ion. Each arrow indicates a magnified view of the area inside the gray square.

**Figure 3:**
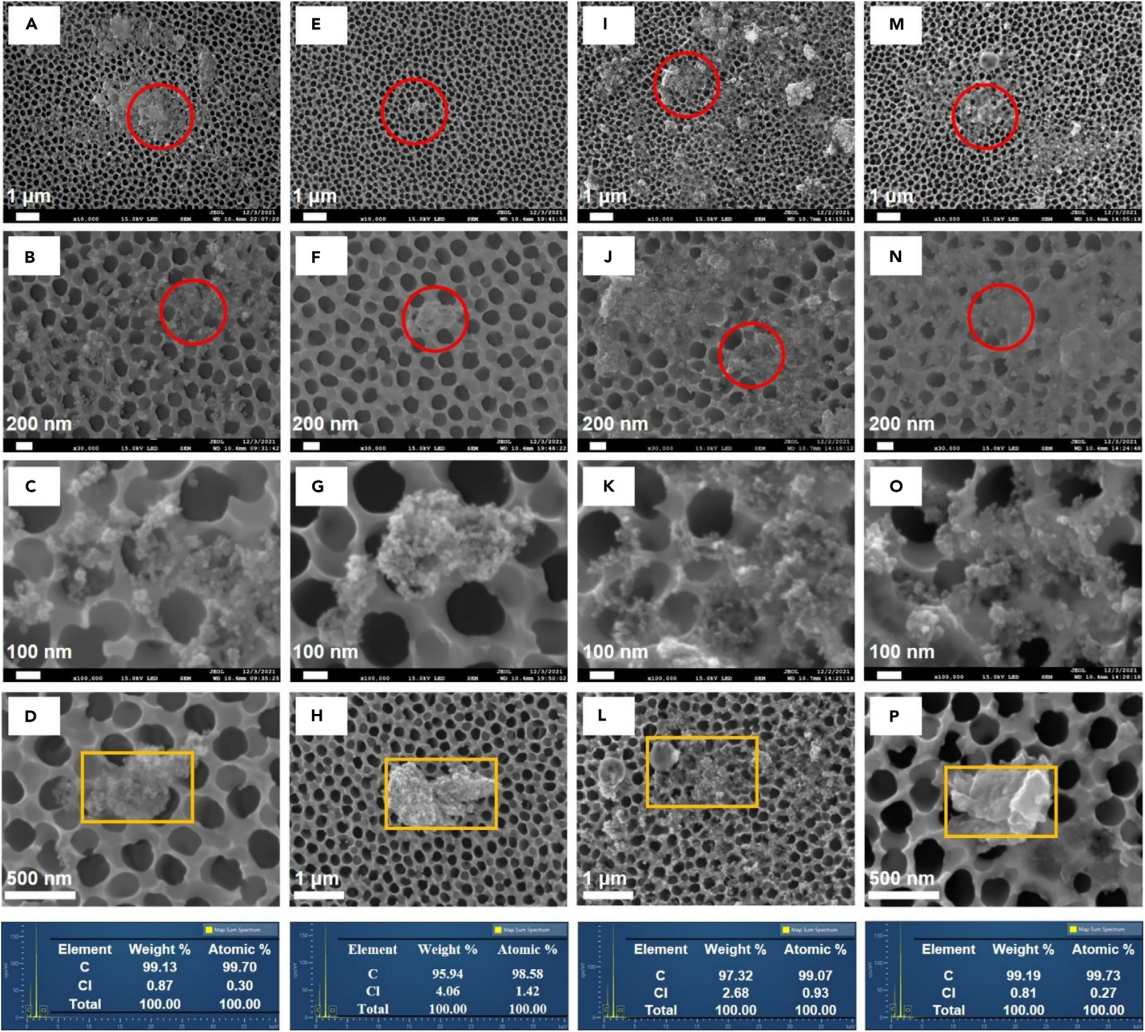
Typical SEM-EDS images of released PVC-MNPs from PVC-IVIPs (A‒D) SEM-EDS images of collected PVC-MNPs by filtering 0.9% sodium chloride injection (normal saline, NS) packed in polyvinyl chloride (PVC) bags. (E‒H) SEM-EDS images of collected PVC-MNPs by filtering NS packed in non-PVC multilayer co-extrusion film infusion bag (NPVC). (I‒L) SEM-EDS images of collected PVC-MNPs by filtering 5% glucose injection (GI) packed in PVC bags. (M‒P) SEM-EDS images of collected PVC-MNPs by filtering GI packed in NPVC. B, F, J, and N represent a magnified view of the red circle in A, E, I, and M, respectively; C, G, K, and O represent a magnified view of the red circle in B, F, J, and N, respectively.

The released MNPs from PVC-IVIPs during the mimic intravenous infusion process were first identified by the previously established methods (Figure 2). Comparing the selected indicator-ion chromatograms and mass spectrum of released MNPs with those of standard PVC (Figure 2B), all the nine pyrolysis products of PVC can be identified with high similarities in their corresponding mass spectra and peak times, suggesting that the released MNPs were PVC-MNPs. Meanwhile, SEM characterization revealed that the released MNPs were spherical and irregular particles with diameters ranging from 1.76 to 83.14 nm (Figure S13). Interestingly, the small particles are present in the form of agglomerates with sizes over 20 nm, and thus were trapped by Al_2_O_3_ membranes (Figure 3). All these MNPs were composed of C and Cl and in agreement with that of standard PVC (Figures 3D, 3H, 3L, and 3P), also suggesting that these MNPs are PVC-MNPs. These results confirmed that the released MNPs are PVC-MNPs, which should originate from PVC-IVIPs because there were no other potential sources. Furthermore, SEM images showed that on the inner surfaces of IVIPs there were some small particles (Figure S14), which could be the main sources of the released PVC-MNPs during intravenous infusion. Taken together, these findings demonstrated that PVC-MNPs can be released from PVC-IVIPs during intravenous infusion.

**Figure 4:**
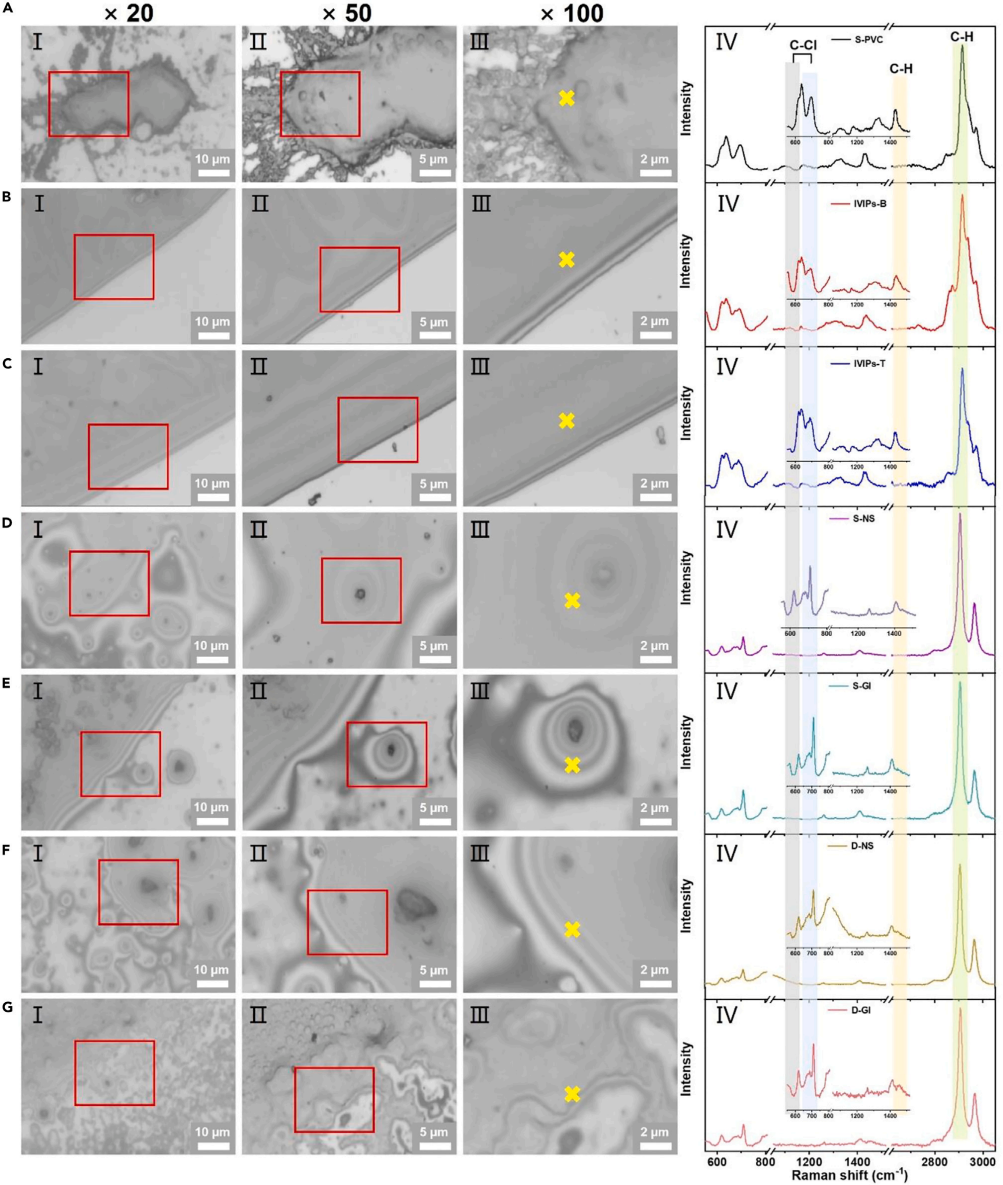
Optical images and Raman spectra of released PVC-MNPs from PVC-IVIPs Samples collected on Al_2_O_3_ membranes were extracted into chloromethane, and dropped on the silicon wafer to form a thin film for Raman detection. (A‒C) Standard PVC (S-PVC), PVC infusion bags (IVIPs-B), and PVC infusion tubes (IVIPs-T) were directly dissolved into dichloromethane; (D and E) 0.5 μg standard PVC-MNPs were spiked in 500 mL of 0.9% sodium chloride injection (normal saline, NS) and 5% glucose injection (GI), respectively, and trapped by filtering with Al_2_O_3_ membranes; (F and G) 500 mL NS and GI were packed into the PVC bags, respectively, followed by passing through the PVC tubes and filtering with Al_2_O_3_ membranes. Optical microscopy images of Ⅰ, Ⅱ, and Ⅲ were obtained under an objective lens of 20×, 50×, and 100×, respectively. In A‒G, Ⅱ represents a magnified view of red box in Ⅰ, and Ⅲ represents a magnified view of red box in Ⅱ. Raman spectra of Ⅳ were obtained at the marked positions in Ⅲ. Inserts in Ⅳ were the magnified view of spectra in the range of 550∼1520 cm^−1^ in A–G.

Raman spectroscopy was also employed to obtain the direct spectral evidence of PVC-MNPs released from PVC-IVIPs during intravenous infusion. Because of their small sizes and amounts as described previously, these PVC-MNPs can hardly be detected directly by Raman spectroscopy and surface-enhanced Raman scattering.^48^ Thus, the PVC-MNPs were extracted by dissolving in dichloromethane (DCM)^52^^,^^53^ and dropped on the silicon wafer, where they present as a thin film after DCM evaporation (Figure 4), facilitating Raman analysis. Figures 4A‒4C show that the characteristic absorption peaks of standard PVC and PVC-IVIPs appeared approximately at 635, 690, 1172, 1435, and 2913 cm^−1^, which remained identical to that before treating by DCM. The absence of typical peaks of PVC in the DCM control indicated that DCM did not interfere with the identification of PVC by Raman spectroscopy (Figure S15B). Therefore, DCM was selected to extract PVC-MNPs from Al_2_O_3_ membranes for acquiring the spectral evidence of released PVC-MNPs. Thin films were observed in both spiked and simulated intravenous infusion samples (Figures 4D‒4G), while irregular blocks rather than typical films were present in the procedural and Al_2_O_3_ membranes blanks (Figures S15C‒S15E). All the characteristic absorption peaks of PVC polymer were detected in these thin films, including approximately 620 and 709 cm^−1^ corresponding to the C−Cl stretching vibration, 2913 cm^−1^ assigned to the C−H stretching vibration, and 1410 cm^−1^ assigned to the C−H stretching vibration and C−H bending vibration (Figures 4D‒4G). In contrast, no typical characteristic absorption peaks of PVC polymer can be obtained in the procedural and Al_2_O_3_ membranes blanks (Figures S15C‒S15E). These findings demonstrate that Raman spectroscopy combined with DCM dissolution can be used to identify PVC-MNPs with masses less than 1 μg, which makes up for the fact that Py-GC-MS can only identify PVC above 1 μg. These results also confirmed the occurrence of PVC-MNPs in simulated intravenous infusion samples, and were the direct evidence of the released PVC-MNPs from PVC-IVIPs during intravenous infusion.

### Quantification of MNPs released from PVC-IVIPs

**Figure 5:**
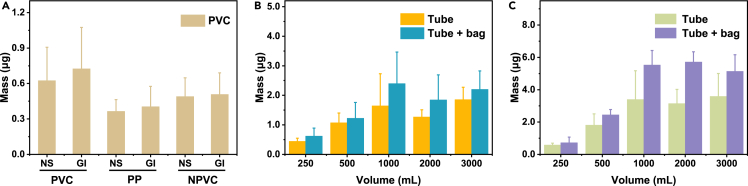
Quantification of released PVC-MNPs from PVC-IVIPs (A) Masses of released PVC-MNPs by treating with 250 mL injections. NS, 0.9% sodium chloride injection (normal saline); GI, 5% glucose injection; PVC, injections were packed by polyvinyl chloride (PVC) bags; PP, injections were packed by polypropylene (PP) bottles; NPVC, injections were packed by non-PVC multilayer co-extrusion film infusion bag (NPVC); (B) Masses of released PVC-MNPs released from tubes and bags, by treating with different volumes of NS. (C) Masses of released PVC-MNPs released from tubes and bags, by treating with different volumes of GI. In B and C, “Tube” represents that the compositions of infusion tubes are PVC, but the filtered injections were packed by non-PVC multilayer co-extrusion film infusion bag (NPVC); “Tube + bag” represents that the compositions of both infusion bag and infusion tubes are PVC. In each figure, the data were obtained by at least three independent experiments and expressed as “mean ± SD” (n ≥ 3).

The released PVC-MNPs from different IVIPs were quantified based on the prepared calibration curves (Figure S12) and proposed experimental program (Table S5). Results showed that the quantities of released PVC-MNPs ranged from 0.36 ± 0.10 to 0.72 ± 0.35 μg (Figure 5A) for all the studied injection types and packages (details in Note S4), indicating that these factors had limited effects on the PVC-MNPs release under this injection volume (250 mL). Specifically, the amounts of released PVC-MNPs by the injections packed in PVC were slightly higher than that packed in PP and NPVC (0.67 μg vs. 0.44 μg). This is reasonable as the released PVC-MNPs could be derived from both PVC tubes and PVC bags, whereas it can only be sourced from PVC tubes in the case of injections packed in PP and NPVC. In other words, the components of IVIPs are the main factors affecting the release of PVC-MNPs from IVIPs. For injection volumes ranged from 250 to 3000 mL in each intravenous infusion, the average masses of released PVC-MNPs were from 1.88 ± 1.14 to 2.78 ± 1.96 μg, which would directly enter human the blood system (Table S6, details in Note S5).

### Influence of injection composition and volume on the MNPs release

We then evaluated the injection composition and volume of intravenous infusion on the release of PVC-MNPs. NPVC packed with the two commonly used NS and GI injections, respectively, were used as representatives to study the release of PVC-MNPs, from both PVC tubes alone and together with bags at the common infusion volume ranging from 250 to 3,000 mL. Figure 5B and C show that the amounts of released PVC-MNPs increased by approximately 3.7–7.6 times with the increase of injection volume from 250 to 1,000 mL, and then level off with further increasing of injection volume to 3000 mL. This result suggests that the particles on the surface of the infusion sets were released in the first injection volume of 1,000 mL; further increase of injection volume released no more PVC-MNPs (details in Note S6). Thus, the ISO recommended injection volume of 500 mL for quality inspection of infusion sets^40^ might underestimate the released MNPs, and the use of 1,000 mL injection is recommended.

Figure 5B and C also show that the amount of released PVC-MNPs from IVIPs by GI injection was larger than that by NS injection (see details in Note S7). This might be ascribed to the fact that GI consists of glucose (5%, m/v), which can provide hydrophobic C–H surface and thus enhanced affinity for hydrophobic compounds in aqueous media,^54^ accelerating the release of hydrophobic PVC-MNPs from the IVIPs. These results confirmed that the injection composition markedly affected the contents of PVC-MNPs released from IVIPs during intravenous injection. However, current standard methods for quality inspection of infusion sets recommended by ISO chose distilled water to test the released particles,^40^ which could underestimate the amounts of released particles. To better evaluate the real release of PVC-MNPs, we propose to use NS, GI, and other commonly used injections to assess the real release of MNPs in quality inspection of infusion sets.

### Particle numbers of PVC-MNPs directly entering human blood circulatory system by intravenous infusion

**Figure 6:**
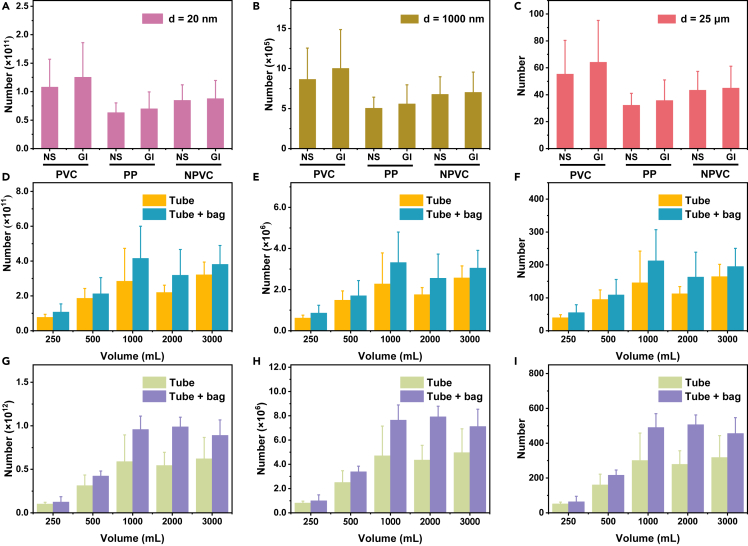
Particle numbers of PVC-MNPs released from PVC-IVIPs (A‒C) Injection volume was 250 mL, see Figure 5A for the meaning of each symbol; (D–F) Injections were 0.9% sodium chloride injection (normal saline, NS), see Figure 5B for the meaning of each symbol; (G‒I) Injections were 5% glucose injection (GI), see Figure 5C for the meaning of each symbol. A, D, and G, assuming the diameter of PVC-MNPs is 20 nm, which is the lowest size collected by this method; B, E, and H, assuming the diameter of PVC-MNPs is 1000 nm, which is the critical size of nanoplastics; C, F, and I, assuming the diameter of PVC-MNPs is 25 μm, which is the lowest size of particles determined by current standard methods.^40^ In each figure, the particle numbers of released PVC-MNPs were calculated based on their quantities, density, and sizes, and assuming all the particles are spherical. The data were obtained by at least three independent experiments and expressed as “mean ± SD” (n ≥ 3).

We further evaluated the potential entry of PVC-MNPs in particle numbers to human blood circulatory system for each intravenous infusion. The particle number concentration of MNPs is a key parameter for evaluating their risks on surrounding environments, living creatures, and even human health.^55^ However, counting the particle number of MNPs is a great challenge due to the lack of efficient analytical techniques.^48^^,^^56^ Here, the particle number of PVC-MNPs was estimated by Equation 2 (shown in STAR Methods) based on their mass, density, and diameter (details in Note S8). For the average amount of released PVC-MNPs (0.52 μg) under the injection volume of 250 mL as determined previously, the particle numbers were calculated as (8.98 ± 2.34) × 10^10^, (7.19 ± 1.87) × 10^5^, and 46 ± 12 by assuming their diameters were 20 nm, 1,000 nm, and 25 μm, respectively (Figures 6A‒6C). If the diameter of released PVC-MNPs was ≥25 μm, the particle number should be ≤46 ± 12 particles, which is less than 90, that is, the maximum permissible number of particles released from IVIPs according to ISO.^40^ However, as the majority of the released PVC-MNPs observed in this study were NPs with diameters <1,000 nm (Figures 3 and S13), the real particle number should be well above (7.19 ± 1.87) × 10^5^. Considering the common infusion volume is ranging from 250 to 3,000 mL in actual intravenous infusion, the potential entry numbers of PVC-MNPs to human blood for each intravenous infusion would range from (2.60 ± 1.57) × 10^6^ to (4.82 ± 3.39) × 10^11^ NPs assuming a diameter of 20–1,000 nm (Table S7, details in Note S8). Figure 6 shows the estimated numbers of released PVC-MNPs under various scenarios, indicating about 10^5^–10^11^ PVC-MNPs could be directly introduced into human blood circulatory system by an intravenous infusion of only 250 mL injections. These results revealed a new exposure route for MNPs to directly enter human blood circulatory system through intravenous infusion, which might be one of the explanations for the occurrence of MPs in human blood reported recently. It should be noted that the aforementioned determination and calculation could underestimate the actual entry contents of MNPs derived from IVIPs during intravenous infusion, as the MNPs other than PVC-MNPs released from other IVIPs such as PP injection bottles and NPVC injection bags or of yet other polymer materials have not been taking into account.

### Implications

This study demonstrated that MNPs can directly enter the human blood circulatory system via intravenous infusion which could be an additional serious route of MNPs to human that has yet been overlooked. Previous studies have indicated that ingestion and inhalation are the primary pathways of human exposure to MNPs;^21^ however, most of these entering MNPs can be excreted out via feces.^20^^,^^57^ This indicated that the bioavailability of these MNPs is low, and thus reducing their potential risks on human health. There is no evidence available on whether these MNPs can be transported into the human blood or other tissues,^58^ but the presence of MNPs in human tissues have been observed. Therefore, previous pathways of ingestion and inhalation cannot explain the sources of MNPs in human blood or other tissues. Unlike the previously reported exposure routes, human exposure to MNPs via intravenous infusion can directly transport MNPs into the human blood, and these MNPs are difficult to excrete and degrade in human blood. Furthermore, the MNPs can be gradually accumulated in human blood and transported to heart, brain, and other important organs along with the human blood circulatory system, which potentially are related to cerebral thrombosis, cardiovascular disease, and other severe diseases.^10^^,^^12^ Thus, human exposure to MNPs via intravenous infusion may well explain the reasons of MNPs detected in human blood or other tissues, and can induce more serious impacts on human health. In addition, MNPs in human blood could undergo a series of processes including aggregation, release additives, non-intentional added substances, and interaction with other compounds, which may affect the toxicity of MNPs on human health. Therefore, further studies are urgently needed to assess the negative effects of the MNPs released from IVIPs during intravenous infusion on human health.

### Conclusion

We have established analytical methods to identify and quantify MNPs down to 20 nm using Py-GC-MS, Raman, and SEM-EDS techniques. The methods were used to identify the release of PVC-MNPs from IVIPs and to quantify the potential particle numbers of PVC-MNPs directly entering human blood during intravenous infusion. We also found that both injection volumes and compositions affected the release of PVC-MNPs from IVIPs during intravenous infusion, and current methods for quality inspection of infusion sets recommended by ISO could underestimate the risk of particle exposure to humans. This study also revealed a previously unknown but important pathway of MNP exposure, which could directly enter human blood circulatory system and cause potential hazards to human health. Further research is necessary to evaluate the potential risks of these MNPs on human health.

### Limitations of the study

We developed a comprehensive analytical method to identify and quantify PVC-MNPs released from intravenous infusion products. The direct entry of MNPs into the human blood system via intravenous infusion was demonstrated in this work. However, this work only took PVC-IVIPs into account but without detecting MNPs released from other IVIPs such as PP injection bottles and NPVC injection bags or of yet other polymer materials, which possibly underestimate the actual entry contents of MNPs derived from IVIPs during intravenous infusion. Future research should determine all the potential MNPs released from IVIPs to comprehensively assess human exposure to MNPs via intravenous infusion. On the other hand, the toxic effects of these MNPs on human health are not investigated, and future studies should focus on animal tests to clarify their impacts on organisms and even human health.

## STAR★Methods

### Key resources table

**Table:** 

REAGENT or RESOURCE	SOURCE	IDENTIFIER
**Chemicals, peptides, and recombinant proteins**		

Ethanol ≥99.7%	Aladdin	CAS 64-17-5
Dichloromethane 99.9%	Energy Chemical	CAS 75-09-2
Polyvinyl chloride particles	Shanghai	PVC-0030N
Polyvinyl chloride infusion tubes	Nanchang	-
Polyvinyl chloride infusion bags	Nanchang	250 mL
Al_2_O_3_ Membrane Filters, 0.02 mm, 25 mm	Whatman	Anodisc™25

**Critical commercial assays**		

Sodium chloride injection packed by polypropylene bottles	Liaocheng	0.9%, 250 mL
Sodium chloride injection packed by polypropylene bottles)	Liaocheng	0.9%, 500 mL
Glucose injection packed by polypropylene bottles	Liaocheng	5%, 250 mL
Glucose injection packed by polypropylene bottles	Liaocheng	5%, 500 mL
Sodium chloride injection packed by non-PVC multilayer co-extrusion film infusion bag	Jining	0.9%, 250 mL
Sodium chloride injection packed by non-PVC multilayer co-extrusion film infusion bag	Jining	0.9%, 500 mL
Glucose injection packed by non-PVC multilayer co-extrusion film infusion bag	Jining	5%, 500 mL
Glucose injection packed by non-PVC multilayer co-extrusion film infusion bag	Jining	5%, 500 mL

**Software and algorithms**		

OriginPro 2019	OriginLab Corporation	Massachusetts, USA

**Other**		

Scanning electron microscopy equipped with energy-dispersive X-ray spectroscopy	FEI	Quanta 250 FEG
Raman spectrometer	Renishaw Invia	inVia
Multi-shot pyrolyzer	Frontier	EGA/PY-3030D
Gas chromatography	Agilent	Agilent-7890A
Mass spectrometry detector	Agilent	Agilent 5975C
Nitrogen evaporator	Organomation	N-EVAP 112

### Resource availability

#### Lead contact

Further information and requests for resources and reagents should be directed to and will be fulfilled by the Lead Contact, Professor Jingfu Liu (liujf@sustech.edu.cn)

#### Materials availability

This study did not generate new unique reagent.

#### Data and code availability

• The published article includes all datasets generated or analyzed during this study.
• Any additional information required to reanalyze the data reported in this paper is available from the lead contact upon request.
• This paper does not report original code.

### Method details

#### Materials

PVC infusion tubes and PVC infusion bags (250 mL) were purchased from Jiangxi Hongda Medical Equipment Group Ltd (Nanchang, China). Both 0.9% sodium chloride injection (normal saline, NS) and 5% glucose injection (GI) packed by polypropylene (PP) bottles with different specifications (250 mL and 500 mL) were obtained from Shangdong Hualu Pharmaceutical Co. Ltd. (Liaocheng, China). The same specifications of NS and GI packed by non-PVC multilayer co-extrusion film infusion bag (NPVC) were bought from Cisen Pharmaceutical Co., Ltd. (Jining, China). Here, we chose NS and GI in the experiments of simulated intravenous infusion as they are the most commonly used injections. Standard PVC particles were purchased from Shanghai YoungLing Technology Co. Ltd. (Shanghai, China). The Al_2_O_3_ membranes with 20 nm pore size and 2.5 cm diameter (Anodisc, Whatman) were used to filter the injections and thus collect PVC-MNPs released from PVC-IVIPs.

#### Characteristics of standard PVC and PVC-IVIPs

Considering the PVC-MNPs released from PVC-IVIPs are at trace level, their mass should be much less than that of the smallest plastic particles that can be cut from PVC-IVIPs. Thus, PVC-MNPs cut from PVC-IVIPs are unsuitable to act as standard substances, and standard PVC-MNPs were used as standards to establish analytical methods. Raman spectroscopy and Py-GC-MS measurements were employed to verify that PVC-IVIPs and standard PVC shared similar components. Raman spectrums of PVC-IVIPs and standard PVC were recorded by a Raman spectrometer (Renishaw Invia, Gloucestershire, UK). Py-GC-MS was performed with a multi-shot pyrolyzer (Frontier, EGA/PY-3030D, Fukushima-ken, Japan) on-line coupled with a GC (Agilent 7890A, Palo Alto, USA) and an MS detector (Agilent 5975C, Palo Alto, USA). Briefly, about 10 μg of small particles were obtained by cutting PVC-IVIPs using a stainless-steel scissor and directly transferred into a deactivated stainless-steel pyrolysis cup for the following Py-GC-MS measurement with operating parameters (Table S1). The deactivated stainless-steel pyrolysis cups were burned with an alcohol lamp for 30 seconds and then covered with aluminum foil to avoid possible contamination prior to use. On the other hand, 0.01 g standard PVC and 9.98 g ethanol were added into a glass test tube, and ultrasonically dispersed for 40 min after sealing to obtain 1000 μg/g standard PVC dispersions. Then, 0.01 g of the dispersions were transferred into a deactivated stainless-steel pyrolysis cup, subsequently dried at 60°C for 40 min, and eventually analyzed by Py-GC-MS. Because NS and GI injections packed by PVC were difficult to obtain in the market, injections packed by PP or NPVC were used as the sources of injections in this study. Thus, both PP bottles and NPVC bags were analyzed by Py-GC-MS using the same procedure as that for PVC-IVIPs to verify if these two-package materials release PVC-MNPs.

#### Development and validation of analytical methods

To develop analytical methods for the determination of PVC-MNPs, the pyrolysis products of different amounts of standard PVC were first analyzed by Py-GC-MS to investigate the relationship between masses of standard PVC and their pyrolysis products. This was applied to determine the smallest quantities that can be used to establish a qualitative method and to select the optimum indicator compound for quantify PVC-MNPs released from PVC-IVIPs. Briefly, proper volume of the standard PVC dispersions was transferred into different pyrolytic target cups and dried at 60°C to obtain standard samples containing 10 ng ∼ 20 μg PVC for Py-GC-MS measurements. According to the relationship between masses of standard PVC and their pyrolysis products, proper quantities of standard PVC were spiked into NS and GI to conduct recovery experiments. Taking spiking of standard PVC into NS as an example, about 1.21 μg and 11.77 μg of standard PVC were spiked into 500 mL of NS, respectively, followed by filtration through a 20 nm pore sized Al_2_O_3_ membrane. Then, the Al_2_O_3_ membrane was dried at 60°C for 2 h, subsequently ground with an agate mortar and transferred to a deactivated stainless-steel pyrolysis cup for Py-GC-MS measurements. For the qualitative method, all nine pyrolysis products of PVC can be observed in the recovery experiments, indicating that the method can be used to identify PVC-MNPs released from IVIPs. For the quantitative method, calibration curves for quantify PVC-MNPs released from IVIPs were prepared by spiking concentrated standard PVC into a deactivated stainless-steel pyrolysis cup, subsequently dried at 60°C for 40 min, and eventually analyzed by Py-GC-MS, respectively. According to the results of Py-GC-MS measurements, the quantitative standard curve was established based on the peak area of benzene (m/z 78) against the masses of standard PVC, which enables the calculation of spiked recoveries. The NS and GI packed by PP bottles (500 mL) without spiking of PVC were also filtered by Al_2_O_3_ membranes to correct procedural contamination of the injection, respectively. Each experiment was repeated at least three times. Recovery (%) is calculated based on Equation 1 as follows:

(Equation 1) $$R\left(\%\right)=\frac{m_{d}}{m_{0}}\times 100\%$$

where *R* (%) represents the recovery of spiked PVC, *m*_*0*_ (g) represents nominal mass of spiked PVC, and *m*_*d*_ (g) is the determined mass of spiked PVC, respectively. Based on the spiked recovery, a quantitative analytical method for PVC-MNPs released from IVIPs was established.

Considering that PVC particles consists of C, H, and Cl elements, SEM-EDS (FEI, Quanta FEG250, Hillsboro, USA) analysis was adopted to characterize the morphology and element composition of the released particles. In brief, the spiked PVC was collected on the Al_2_O_3_ membranes, dried by vacuum, then gold sprayed for 120 seconds, and eventually used for SEM-EDS to verify morphologically that the spiked PVC was indeed collected by Al_2_O_3_ membranes. In addition, the inner surfaces of original PVC-IVIPs were also determined by SEM for disclosing the potential sources of released PVC-MNPs. The size distribution of standard PVC-MNPs was obtained by counting at least 200 particles with Nano Measure 1.2 software.

#### Quality control

Considering that the quantitative indicator compound is benzene (m/z 78), which is easily contaminated from the surroundings, strict procedure blanks were conducted to avoid potential contamination from air, experimental materials and procedures. To prepare the procedure blank for evaluating potential contamination from air, the Al_2_O_3_ membranes without injections was placed in the filter for the identical time as the simulated infusion process. To obtain the procedural blanks in the identification and quantification of the released PVC from IVIPs, the injections of NS/GI were directly transferred into the filter without going through the infusion tubes. Subsequently, these Al_2_O_3_ membranes were transferred into glass petri dishes covered with aluminum foil and dried for Py-GC-MS determination, respectively. All the glass containers were washed with ultrapure water and then covered with aluminum foil until the next use to avoid contamination from the surroundings.

#### Identification of MNPs released from PVC-IVIPs

Simulation of intravenous infusion process in the lab was conducted to identify PVC-MNPs released from PVC-IVIPs. Briefly, about 3000 mL NS or GI were transferred into PVC bags, subsequently flowed through the PVC infusion sets at the rate of 40∼60 drops/min that is commonly adopted in real intravenous infusion, and finally filtered with the Al_2_O_3_ membranes to collect the potential PVC-MNPs released from PVC-IVIPs. Then, the Al_2_O_3_ membranes were dried and used for Py-GC-MS measurements as mentioned above. According to the pyrolysis products determined by Py-GC-MS, identification of PVC-MNPs released from PVC-IVIPs can be achieved. In order to obtain the morphology of the potential PVC-MNPs released from PVC-IVIPs, another simulated intravenous infusion experiments were also conducted to collect PVC-MNPs on Al_2_O_3_ membranes and used for SEM-EDS analysis.

To acquire the direct spectral evidence of the released PVC-MNPs with low concentration (<1 μg) from PVC-IVIPs during intravenous infusion, the PVC-MNPs collected on the membrane were extracted by DCM and detected by Raman spectroscopy according to previous studies with some modifications.^52^^,^^53^ To verify that standard PVC-MNPs and PVC-IVIPs used in this work can be dissolved in DCM, these PVC materials were directly dissolved into DCM and used for Raman measurements. Specifically, into a glass bottle containing 5 mL DCM was added 0.5 μg standard PVC-MNPs, and the mixture was ultrasound at 60°C for 2 h and then concentrated using a nitrogen evaporator (N-EVAP 112, USA) to several drops, which was dropped on a silicon wafer for Raman analysis. Debris of pristine PVC infusion bags (IVIPs-B) and PVC infusion tubes (IVIPs-T) were also added into DCM and treated with the same procedure as mentioned above, respectively. About 10 μL DCM were directly dropped on a silicon wafer to investigate whether DMC affected the identification of PVC based on Raman analysis.

Spiked experiments were conducted to verify whether PVC-MNPs collected on Al_2_O_3_ membranes can be extracted by DCM. About 0.5 μg standard PVC were added into 500 mL NS and GI, and then directly collected on Al_2_O_3_ membranes (detailed in section of development and validation of analytical methods), respectively. Procedural experiments were also conducted (detailed in section of quality control) to exclude the influence of injection solutions on the identification of PVC-MNPs. As for the potential PVC-MNPs released from PVC-IVIPs, another simulated intravenous infusion experiments were conducted as mentioned above to obtain the released PVC-MNPs collected on Al_2_O_3_ membranes. The dried Al_2_O_3_ membranes were transferred into a glass bottle by a stainless-steel tweezer, followed by adding 10 mL DCM, and then ultrasound at 60°C for 2 h. Subsequently, the supernatant was transferred into a new glass bottle. Meanwhile, the residual Al_2_O_3_ membranes were washed with DCM (5 mL) for three times and the supernatant were also transferred into the same glass bottle, and concentrated to a few drops using a nitrogen evaporator. Eventually, 10 μL residues were dropped on a silicon wafer and dried for Raman analysis. Additionally, pristine Al_2_O_3_ membranes were also treated as samples to investigate their effects on the identification of the potential PVC-MNPs released from IVIPs. Raman spectra of all the samples were collected at 532 nm laser excitation (laser power, 50%) with an integration time of 10 s under an objective lens of 100×, and a silicon wafer was applied to calibrate the Raman spectrometer prior to analysis.

#### Quantitation of PVC-MNPs released from PVC-IVIPs

To quantitatively determine PVC-MNPs released from PVC-IVIPs, the released PVC-MNPs were collected on Al_2_O_3_ membranes and then analyzed by Py-GS-MS using the same experimental procedures as that for identifying PVC-MNPs released from PVC-IVIPs. Considering that medical infusion products are generally packed with PVC, PP and NPVC, and medical infusion tube is commonly made of PVC, batch experiments were conducted to measure PVC-MNPs released from these IVIPs. Then, two experimental groups, including NS/GI + PVC bags + PVC tubes and NS/GI + NPVC bags + PVC tubes, were selected to investigate the effect of infusion composition (NS or GI) and volume (250, 500, 1000, 2000, and 3000 mL) on the release of PVC-MNPs from IVIPs.

#### Evolution of particle number of released PVC-MNPs

The particle number of PVC-MNPs was calculated by assuming the particles are spherical based on Equation 2:

(Equation 2) $$N=\frac{m_{p}}{m_{e}}=\frac{{6m}_{p}}{{\pi \rho d}^{3}}$$

where *m*_*e*_ (g) represents the masses of each PVC-MNPs particle; *m*_*p*_ (g) is the mass of total released PVC-MNPs obtained by Py-GC-MS measurements; *ρ* is the density of PVC (1.38 g/cm^3^) provided by the manufacturers; π is a constant (3.14); and *d* (cm) is the diameter of the released PVC-MNPs.

### Quantification and statistical analysis

All the results were the average of tri-measurements with standard error.

## Supplemental information

Document S1. Figures S1‒S15, Tables S1‒S7, and Notes S1−S8
