# Supplementary material for: Direct entry of micro(nano)plastics into human blood circulatory system by intravenous infusion
Source: iScience. 2023 Nov 14;26(12):108454. doi: 10.1016/j.isci.2023.108454 (PMC10709129; doi:10.1016/j.isci.2023.108454)
Supplement: Document S1. Figures S1‒S15, Tables S1‒S7, and Notes S1−S8 [file mmc1.pdf]

iScience, Volume *26*

## Supplemental information

# Direct entry of micro(nano)plastics into human blood circulatory system by intravenous infusion

**Penghui Li, Qingcun Li, Yujian Lai, Shuping Yang, Sujuan Yu, Rui Liu, Guibin Jiang, and Jingfu Liu**

# Supplementary Information

Summary

Number of Pages: 30


Pages 2−7: Supplementary Notes 1−8

Pages 8−13: Supplementary Tables. 1−7

Pages 14−28: Supplementary Figures. 1−15

Pages 29−30: Supplementary References

**Supplementary Note 1. Comparisons of the main pyrolysis products of different intravenous infusion products (IVIPs). Related to the section of Characterization of standard PVC and pristine PVC-IVIPs**

Polyvinyl chloride (PVC), polypropylene (PP), and non-PVC multilayer co-extrusion film (NPVC) are the typical materials commonly used for producing intravenous infusion products (IVIPs)[1, 2]. Considering that the presence of PP and NPVC particles may influence the determination of PVC micro(nano)plastics (PVC-MNPs), pyrolysis-gas chromatography-mass spectrometry (Py-GC-MS) was used to analyze and compare the main pyrolysis products of PVC-IVIPs with that of PP- and NPVC-IVIPs. Results showed that the main pyrolysis products of PVC-IVIPs were aromatic molecules, including benzene, methylbenzene, styrene, indene, methyl indene, naphthalene, methylnaphthalene, acenaphthene and anthracene (**Figure S4, S6, Table S2**), which were in good agreement with pyrolysis products of standard PVC[3]. The main pyrolysis products of PP and NPVC IVIPs were aliphatic compounds, such as $C_6$ (2-methyl-1-pentene), $C_9$ (2, 4-dimethyl-1-heptene), $C_{12}$ (2, 4, 6-trimethyl-1-nonene) and $C_{15}$ (2, 4, 6, 8-tetramethyl-1-undecene, as shown in **Figure S4, S7** and **S8**), which was in accordance to pyrolysis products of standard PP[4]. These results indicate that the pyrolysis products of PVC-IVIPs are different from that of PP- and NPVC-IVIPs. Therefore, the injections packed by PP and NPVC had no influences on the determination of PVC-MNPs by Py-GC-MS.

**Supplementary Note 2. Selection of sample mass and indicator ion for Py-GC-MS determination of PVC-MNPs. Related to the section of Characterization of standard PVC and pristine PVC-IVIPs**

To select the suitable mass and indicator ion of PVC for the Py-GC-MS determination, the relationships between the main pyrolysis products and PVC mass were analyzed. Results showed that the pyrolysis products of standard PVC mainly included benzene, methylbenzene, styrene, indene, methyl indene, naphthalene, methylnaphthalene, acenaphthene and anthracene (**Figure S4, S5, Table S2**), which was in good agreement with previous studies[5, 6]. However, the main pyrolysis products were benzene, methylbenzene and styrene when less than 1 μg of PVC mass was analyzed. With PVC mass increasing to ≥ 1 μg, all of the nine pyrolysis products remained stable (**Figure S9**), indicating that PVC mass strongly affected their pyrolysis products. Here, the presence of PVC-

MNPs was identified only when all the nine pyrolysis products of PVC were observed in the samples. These results confirmed that 1 μg was the minimum PVC mass required to effectively identify PVC-MNPs released from PVC-IVIPs through Py-GC-MS. Identification of PVC less than 1 μg can be achieved by Raman spectroscopy detailed in section of **Identification of MNPs released from PVC-IVIPs**. On the other hand, the percentages of each pyrolysis product were also analyzed to select the indicator ion for quantitative determination of PVC-MNPs. Results showed that the percentage of benzene was approximately 70% regardless of PVC mass (**Figure S9**), indicating the benzene was the most abundant and sensitive pyrolysis product of PVC, which facilitates to determine the low concentration of PVC[7-9]. Therefore, benzene (m/z 78) was thus selected as the indicator compound to quantify low masses of PVC-MNPs released from PVC-IVIPs.

**Supplementary Note 3. Quality control on the determination of PVC-MNPs released from PVC-IVIPs. Related to the section of Development and validation of analytical methods for PVC-MNPs**

Considering that the typical pyrolysis products of PVC may also originate from the air, injections and other potential sources, analysis of air blank and strict procedure blanks were conducted for quality control (details in section of **Quality control**). According to the results of air blank, there were no notable pyrolysis products of PVC observed in air blank (**Figure 2**$a_{IV}$), suggesting that the air and $Al_2O_3$ membrane exhibited little influence on the identification of PVC-MNPs. However, many peaks can be detected in both simulated infusion (NS, **Figure 2**$a_{II}$; GI, **Figure 2**$a_{III}$) and procedure blanks (NS, **Figure 2**$a_{V}$; GI, **Figure 2**$a_{VI}$). To determine whether the procedure blanks affected the determination of released PVC, we further extracted and analyzed selected indicator-ion chromatograms (SIC) and mass spectrum (MS) of the target indicator ion, which were then compared with that of standard PVC. Few pyrolysis products of PVC (such as methylbenzene, methylnaphthalene) can be observed in the procedure blanks of NS or GI (**Figure S11**), which probably derived from pyrolysis of some impurities introduced in the production of injections. Even so, these products did not affect the identification of released PVC-MNPs since only parts rather than all of the pyrolysis products of PVC were observed in procedural blanks. Because the presence of PVC-MNPs was confirmed only on condition that all the nine pyrolysis products of PVC were

detected, the procedure blanks thus could have little influence on the identification of PVC-MNPs. For quantifying the released PVC-MNPs, the quantitative indicator compound was selected as benzene (m/z 78), which is easily contaminated from the air, experimental materials and experimental procedure. To guarantee the accuracy of PVC-MNP determination, the PVC mass is calculated based on a calibration curve depending on the corrected peak area of benzene, which is obtained by the peak area of benzene in the samples minus that determined in the procedure blanks. For example, when the NS was used to investigate the released PVC-MNPs from PVC-IVIPs, the corrected peak area of benzene was obtained by using the peak area of benzene in the filtrate of NS minus that in the procedure blank of NS. Therefore, the strict quality control in this work can ensure the accurate determination of PVC-MNPs released from PVC-IVIPs.

**Supplementary Note 4. Masses of the potential PVC-MNPs released from PVC-IVIPs. Related to the section of Quantification of MNPs released from PVC-IVIPs**

The masses of released PVC-MNPs were directly determined based on the developed method through Py-GC-MS analysis. Results showed that the masses of released PVC-MNPs showed an average value of 0.52 ± 0.13 μg, ranging from 0.36 ± 0.10 to 0.72 ± 0.35 μg, which were in the same order of magnitude regardless of the injection types and packages. However, there were also some differences present in the quantities of released PVC-MNPs. The masses of released PVC-MNPs were 0.36 ~ 0.51 μg, which showed no remarkable differences when the injections (250 mL) were packed by PP or NPVC (**Figure 5**a). Considering that PVC-MNPs were unlikely to be released from PP or NPVC materials, PVC tubes might be the only source of released PVC-MNPs, which well explained their similar mass of released PVC-MNPs for two different packages. In contrast, when the injections were packed by PVC, the masses of released PVC-MNPs were 0.62 ± 0.28 μg for NS and 0.72 ± 0.35 μg for GI. These two values were slightly higher than that when the injections were packed by PP and NPVC (**Figure 5**a). This is reasonable as the released PVC-MNPs are derived from not only PVC tubes but also PVC bags. Therefore, components of IVIPs are the main factors affecting the release of PVC-MNPs from IVIPs.

**Supplementary Note 5. Masses of PVC-MNPs directly entering human blood circulatory**

**system by intravenous infusion. Related to the section of Quantification of MNPs released from PVC-IVIPs.**

Considering that both organic and inorganic injections ranging from 250 to 3000 mL can be used in the actual intravenous infusion, we evaluated the masses of released PVC-MNPs from IVIPs during intravenous infusion (**Table S6**). When only a single injection solution (NS or GI) was used in intravenous infusion, the potential masses of PVC-MNPs entering human blood for one intravenous infusion were averaged as 1.26 ± 0.55 μg for NS and 2.51 ± 1.28 μg for GI if only the injection tubes were composed of PVC. By contrast, the average mass of PVC-MNPs entering human blood increased slightly to 1.66 ± 0.73 μg for NS and 3.91 ± 2.22 μg for GI when both injection tubes and bags were made of PVC (**Table S6**). However, both inorganic and organic injection can be used in a certain order because it is unlikely that only a single injection solution was used in the realistic intravenous infusion. Here, NS and GI were on behalf of inorganic and organic injection, respectively. According to the data obtained above, we calculated the masses of released PVC-MNPs in the presence of both inorganic and organic injections, which may be much closer to their real masses entering human blood in one intravenous infusion. Results showed that the average masses of PVC-MNPs directly entering human blood in one intravenous infusion were 1.88 ± 1.14 μg when only PVC tubes were used, and 2.78 ± 1.96 μg when both PVC tube and bag were used (**Table S6**).

**Supplementary Note 6. Influence of injection volume on PVC-MNP release. Related to the section of Influence of injection composition and volume on the MNPs release**

**Figure 5**b, c show that the release of MNPs from IVIPs was influenced by both injection volume and type. For the same injection type, the contents of PVC-MNPs released from PVC-IVIPs increased with the injection volume at the beginning and then remained stable. For NS injection, with the increase of injection volume from 250 to 1000 mL, the PVC-MNPs released only from PVC tubes increased from 0.45 μg to 1.64 μg; whereas the total PVC-MNPs released from both PVC tubes and bags increased from 0.62 μg to 2.40 μg (**Figure 5**b). By replacing NS with GI injections, the release of PVC-MNPs showed the same trends, i.e., increased from 0.58 μg to 3.40 μg for PVC tubes, and from 0.72 to 5.52 μg for PVC tubes and bags together (**Figure 5**c). These

results indicated that the mass of released PVC-MNPs from IVIPs increased about one order of magnitude in both NS (**Figure 5**b) and GI (**Figure 5**c) when the injection volumes increased from 250 mL to 1000 mL, and then remained basically unchanged as the injection volume continued to increase to 3000 mL. Interestingly, the mass of released MNPs from PVC tubes and bags together was slightly higher than that from PVC tubes only at each injection volume, indicating that both PVC tube and bag can release PVC-MNPs and PVC tube is the main source of PVC-MNPs during intravenous infusion.

**Supplementary Note 7. Influence of injection type on PVC-MNPs release. Related to the section of Influence of injection composition and volume on the MNPs release**

Under the same injection volume, the amounts of released PVC-MNPs from IVIPs collected by filtering GI injection (**Figure 5**c) were higher than that of NS injection (**Figure 5**b). For example, the masses of PVC-MNPs released from PVC tubes and bag by 3000 mL GI (5.14 μg) were 2.34 times to that by 3000 mL NS (2.20 μg). Considering that the injection volumes were in the range of 250 to 3000 mL in the real intravenous infusion, the contents of released PVC-MNPs were evaluated according to the data shown in **Figure 5**b, c. As shown in **Table S6**, the average masse of released PVC-MNPs from PVC tubes by GI injection (2.51 ± 1.28 μg) were higher than that by NS injection (1.26 ± 0.55 μg). When the injections were packed by PVC bags, the released amounts of PVC-MNPs by GI and NS were 3.91 ± 2.22 and 1.66 ± 0.73 μg, respectively, also indicating that PVC-IVIPs released more PVC-MNPs by GI injections. These results demonstrated that PVC-MNPs released from PVC-IVIPs in the presence of GI was higher than that by NS. In addition, the masses of PVC-MNPs released form PVC bag and tubes were higher than that released from PVC tubes only, indicating that both PVC tube and bag can release PVC-MNPs.

**Supplementary Note 8. Particle numbers of PVC-MNPs directly entering human blood circulatory system by intravenous infusion. Related to the section of Particle numbers of PVC-MNPs directly entering human blood circulatory system by intravenous infusion**

For the released PVC-MNPs, counting the particle number concentration of MNPs is a challenging task due to the lack of efficient analytical techniques[10, 11]. Here, we assumed that the

released PVC-MNPs were spherical particles with three critical diameters of released PVC-MNPs, including the smallest size of particles collected by this method (20 nm), the critical size of nanoplastics (1000 nm)[12] and the smallest size of particles determined by the current standard methods (25 μm)[13], respectively, to estimate the particle numbers. The particle number of released PVC-MNPs was evaluated based on their mass, density and diameter (**eq. 2**), which were also used to assess the amounts of MNPs in previous studies[14, 15]. For the 250 mL injections, the estimated particle numbers of released PVC-MNPs were in the range of $(6.31 \pm 1.72) \times 10^{10} \sim (1.25 \pm 0.61) \times 10^{11}$, with an average of $(8.98 \pm 2.34) \times 10^{10}$ for the critical size of 20 nm (**Figure 6**a); $(5.05 \pm 1.37) \times 10^{5} \sim (1.00 \pm 0.49) \times 10^{6}$, with a mean of $(7.19 \pm 1.87) \times 10^{5}$ for the critical size of 1000 nm (**Figure 6**b); and 32 ± 9 ~ 64 ± 31, with an average of 46 ± 12, for the critical size of 25 μm (**Figure 6**c). In this regard, the amounts of released PVC-MNPs were less than 46 ± 12 particles if their diameters were larger than 25 μm.

**Table S7** shows the evaluated numbers of PVC-MNPs directly entering human blood circulatory system by different injection volumes during intravenous infusion. For the critical diameter of 25 μm, the average particle numbers of PVC-MNPs directly entering human blood for one intravenous infusion were 167 ± 101 particles from PVC tube only, and 247 ± 173 particles from PVC tube and bag together. For the critical diameter of 1000 nm, the particle numbers of PVC-MNPs ranged from $(0.62 \pm 0.14) \times 10^{6}$ to $(4.97 \pm 1.96) \times 10^{6}$ with an average of $(2.60 \pm 1.57) \times 10^{6}$ particles when only PVC tubes were used, and in the range of $(0.86 \pm 0.37) \times 10^{6} \sim (7.91 \pm 1.42) \times 10^{6}$ with a mean of $(3.86 \pm 2.71) \times 10^{6}$ particles when both PVC tubes and bags were used. When the critical diameter was chosen to be 20 nm, the amounts of released PVC-MNPs for one intravenous infusion would achieve tens of billions, which would be appalling if these particles directly entered human blood. Specifically, the potential entry amounts of PVC-MNPs into human blood for one intravenous infusion would be $(0.77 \pm 0.18) \times 10^{11} \sim (6.21 \pm 2.45) \times 10^{11}$ with an average of $(3.26 \pm 1.97) \times 10^{11}$ particles when only PVC tubes were used, while the numbers would be $(1.07 \pm 0.47) \times 10^{11} \sim (9.89 \pm 1.78) \times 10^{11}$ particles with an average of $(4.82 \pm 3.39) \times 10^{11}$ particles when both PVC tubes and bags were used. Considering that the sizes of PVC-MNPs were mainly less than 1000 nm as observed in **Figure 3** and **Figure S13**, we thus selected 20 nm and 1000 nm as the critical diameters to estimate the most probable numbers of PVC-MNPs entering

the human blood circulatory system. By this way, the mean numbers of PVC-MNPs directly entering human blood circulatory system in one intravenous infusion ranged from (2.60 ± 1.57) × $10^6$ to (3.26 ± 1.97) × $10^{11}$ particles when only PVC tube was used, and from (3.86 ± 2.71) × $10^6$ to (4.82 ± 3.39) × $10^{11}$ particles when both of PVC tube and bag were used.

**Table S1.** Instrumental parameters of the Py-GC-MS system. Related to the STAR Methods.

| **Pyrolyzer** | **Frontier EGA/PY-3030D** | **Mass spectrometer** | **Agilent 5975C** |
|---|---|---|---|
| Carrier gas | Helium | Mode | Full scan |
| Oven temperature | 590°C | Scan time | 0.4 s |
| Interface temperature | 300°C | Ionization energy | 70 eV |
| Pre-purge time | 10 s | Scan rate | 2.48 scans/s |
| Pyrolysis time | 18 s | Scan range | 10-550 amu |
| | | Source temperature | 230°C |
| | | Quadrupole temperature | 150°C |
| **Gas chromatogram** | **Agilent 7890A** | | |
| Split ratio | 30:1 | | |
| Temperature | 300°C | | |
| Column | HP-5MS column; 30 m x 0.25 mm; film thickness 0.25 µm | | |
| Flow | 1 mL/min | | |
| Temperature program | 50°C (2 min) ⟶ 320°C (3 min) | | |
| Transfer line temperature | 280°C | | |

**Table S2.** Typical pyrolysis products of standard PVC. Related to Figure 1c.

| Type | m/z | Peak time (min) |
|---|---|---|
| Benzene | 78 | 1.9 |
| Methylbenzene | 91 | 2.8 |
| Styrene | 104 | 4.6 |
| Indene | 116 | 7.4 |
| Methyl indene | 130 | 9.2 |
| Naphthalene | 128 | 9.6 |
| Methylnaphthalene | 142 | 11.4 |
| Acenaphthene | 154 | 12.5 |
| Anthracene | 178 | 17.2 |

Note: benzene was the most abundant and sensitive pyrolysis product of PVC, and thus selected as the indicator compound to qualify the released PVC-MNPs.

**Table S3.** Analytical performance of the proposed method. Related to Figure 1.

| Species | Linear range (μg) | $R^2$ | RSD (%, n⩾3) | LOD (μg) | LOQ (μg) |
|---|---|---|---|---|---|
| PVC-MNPs | 0.01-20 | 0.9983 | 6.67 | 0.0004 | 0.0012 |

The limit of Detection (LOD) (based on 3 times baseline noise, S/N = 3) and the limit of quantification (LOQ) (based on 10 times baseline noise, S/N = 10)

**Table S4.** Recovery rates of spiked PVC with different qualities in NS and GI. Related to Figure 1.

| **Injection types** | **Spiked PVC (μg)** | **Recovery rate (%)** | **RSD (%, n⩾3)** | **Calibration curves** |
|---|---|---|---|---|
| NS | 1.21 | 83.54 | 12.63 | **Fig S12a** |
| GI | 1.21 | 98.37 | 10.76 | **Fig S12a** |
| NS | 11.77 | 80.23 | 12.03 | **Fig S12b** |
| GI | 11.77 | 86.60 | 10.33 | **Fig S12b** |

NS represents 0.9% sodium chloride injection (normal saline, NS); GI represents 5% glucose injection (GI).

**Table S5.** Experimental program to measure PVC-MNPs released from different medical infusion products. Related to the STAR Methods.

| Experimental programs | Number of samples | Injection types | Volume (mL) | Components of infusion bottles/bags | Components of infusion tubes |
|---|---|---|---|---|---|
| Group 1 | 3 | NS | 250 | PP | PVC |
| Group 2 | 3 | GI | 250 | PP | PVC |
| Group 3 | 3 | NS | 250 | NPVC | PVC |
| Group 4 | 3 | GI | 250 | NPVC | PVC |
| Group 5 | 3 | NS | 250 | PVC | PVC |
| Group 6 | 3 | GI | 250 | PVC | PVC |

NS represents 0.9% sodium chloride injection (normal saline, NS); GI represents 5% glucose injection (GI). NPVC represents non-PVC multilayer co-extrusion film infusion bags. PP and PVC represent polypropylene and polyvinyl chloride, respectively.

**Table S6.** Evaluated masses of PVC-MNPs entering human blood circulatory system by intravenous infusion. Related to Figure 5.

| Injection type | Test parts | Min/μg | Max/μg | Mean/μg |
|---|---|---|---|---|
| NS | Tubes | 0.45 ± 0.10 | 1.86 ± 0.42 | 1.26 ± 0.55 |
| GI | Tubes | 0.58 ± 0.11 | 3.59 ± 1.41 | 2.51 ± 1.28 |
| NS and GI | Tubes | 0.45 ± 0.10 | 3.59 ± 1.41 | 1.88 ± 1.14 |
| NS | Tubes and bags | 0.62 ± 0.27 | 2.40 ± 1.07 | 1.66 ± 0.73 |
| GI | Tubes and bags | 0.72 ± 0.35 | 5.71 ± 1.03 | 3.91 ± 2.22 |
| NS and GI | Tubes and bags | 0.62 ± 0.27 | 5.71 ± 1.03 | 2.78 ± 1.96 |

"NS" represents 0.9% sodium chloride injection (normal saline, NS) on behave of inorganic injection; "GI" represents 5% glucose injection on behave of organic injection; "NS and GI" represents that the injection may be NS or GI in actual intravenous infusion. "Tubes" represent that injection tubes are composed of PVC while injection bags are composed of NPVC; "Tubes and bags" represent that both injection tubes and bags are composed of PVC.

**Table S7.** Evaluated amounts of PVC-MNPs entering human blood by intravenous infusion. Related to Figure 6.

| Injection type | Test part | Diameter | Min/particles | Max/particles | Mean/particles |
|---|---|---|---|---|---|
| | | | $\times 10^{11}$ | $\times 10^{11}$ | $\times 10^{11}$ |
| NS | Tubes | 20 nm | 0.77 ± 0.18 | 3.21 ± 0.73 | 2.18 ± 0.95 |
| GI | Tubes | 20 nm | 1.01 ± 0.20 | 6.21 ± 2.45 | 4.34 ± 2.21 |
| NS and GI | Tubes | 20 nm | 0.77 ± 0.18 | 6.21 ± 2.45 | 3.26 ± 1.97 |
| NS | Tubes and bags | 20 nm | 1.07 ± 0.47 | 4.15 ± 1.85 | 2.87 ± 1.27 |
| GI | Tubes and bags | 20 nm | 1.25 ± 0.61 | 9.89 ± 1.78 | 6.77 ± 3.84 |
| NS and GI | Tubes and bags | 20 nm | 1.07 ± 0.47 | 9.89 ± 1.78 | 4.82 ± 3.39 |
| **Injection type** | **Test part** | **Diameter** | **Min/particles** | **Max/particles** | **Mean/particles** |
| | | | $\times 10^{6}$ | $\times 10^{6}$ | $\times 10^{6}$ |
| NS | Tubes | 1000 nm | 0.62 ± 0.14 | 2.57 ± 0.58 | 1.74 ± 0.76 |
| GI | Tubes | 1000 nm | 0.81 ± 0.16 | 4.97 ± 1.96 | 3.47 ± 1.77 |
| NS and GI | Tubes | 1000 nm | 0.62 ± 0.14 | 4.97 ± 1.96 | 2.60 ± 1.57 |
| NS | Tubes and bags | 1000 nm | 0.86 ± 0.37 | 3.32 ± 1.45 | 2.30 ± 1.01 |
| GI | Tubes and bags | 1000 nm | 1.00 ± 0.49 | 7.91 ± 1.42 | 5.41 ± 3.07 |
| NS and GI | Tubes and bags | 1000 nm | 0.86 ± 0.37 | 7.91 ± 1.42 | 3.86 ± 2.71 |
| **Injection type** | **Test part** | **Diameter** | **Min/particles** | **Max/particles** | **Mean/particles** |
| NS | Tubes | 25 μm | 39 ± 9 | 164 ± 37 | 111 ± 49 |
| GI | Tubes | 25 μm | 52 ± 10 | 318 ± 125 | 222 ± 113 |
| NS and GI | Tubes | 25 μm | 39 ± 9 | 318 ± 125 | 167 ± 101 |
| NS | Tubes and bags | 25 μm | 55 ± 24 | 213 ± 95 | 147 ± 65 |
| GI | Tubes and bags | 25 μm | 64 ± 31 | 506 ± 90 | 346 ± 197 |
| NS and GI | Tubes and bags | 25 μm | 55 ± 24 | 506 ± 90 | 247 ± 173 |

"NS" represents 0.9% sodium chloride injection (normal saline, NS); "GI" represents 5% glucose injection; "NS and GI" represents that the injection may be NS or GI in actual intravenous infusion. "Tubes" represent that injection tubes are composed of PVC while injection bags are composed of NPVC; "Tubes and bags" represent that both injection tubes and bags are composed of PVC. "Diameter" represents the critical diameters of the released PVC-MNPs, including the lowest size collected by this method (20 nm), the critical size of nanoplastics (1000 nm) and the lowest size of particles determined by current standard methods (25 μm).

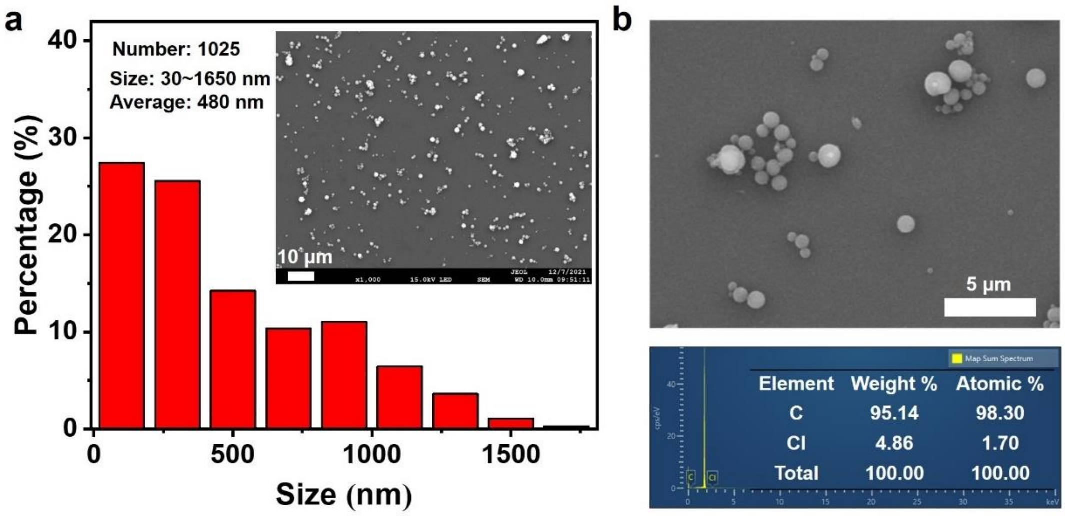


**Figure S1.** SEM analysis of standard PVC particles. Related to Figure 1.

Note: **a**, SEM images and size distributions of standard PVC particles. **b**, SEM-EDS images of standard PVC particles directly dropped on silicon wafer.

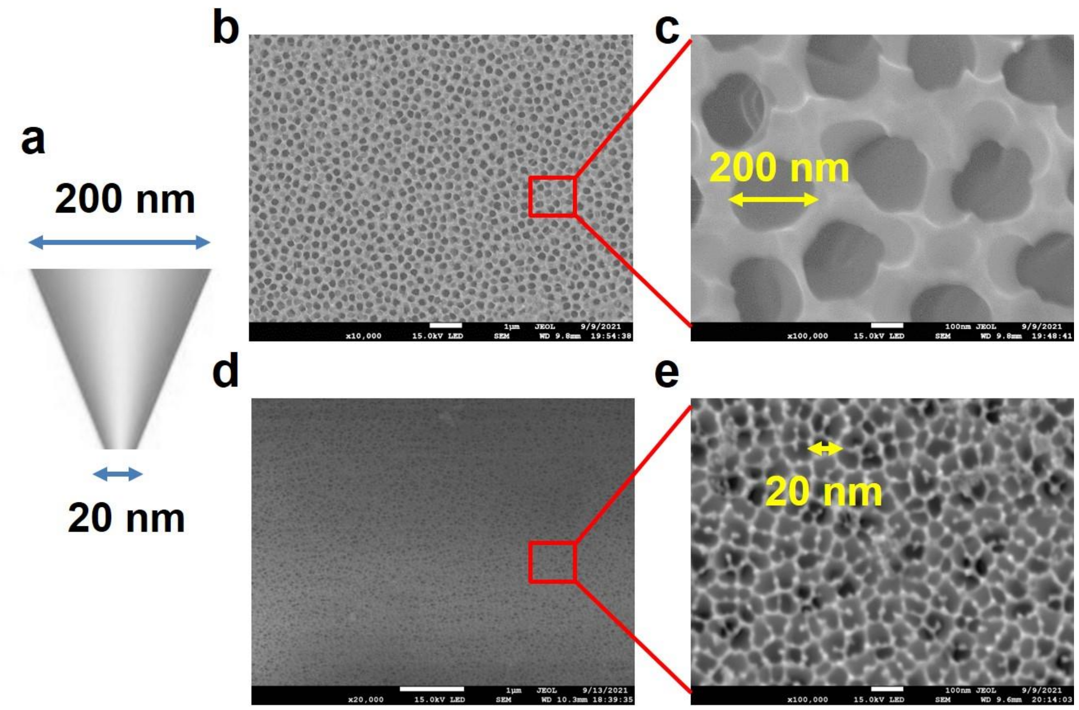


**Figure S2.** Typical SEM images and structure diagram of $Al_2O_3$ filter membranes used in this study. Related to Figure 1.

Note: **a**, the structure diagram of $Al_2O_3$ filter membranes; **b** and **d**, two sides of an alumina membrane, respectively; **c** and **e**, a magnified view of the area inside the red square of **b** and **d**, respectively.

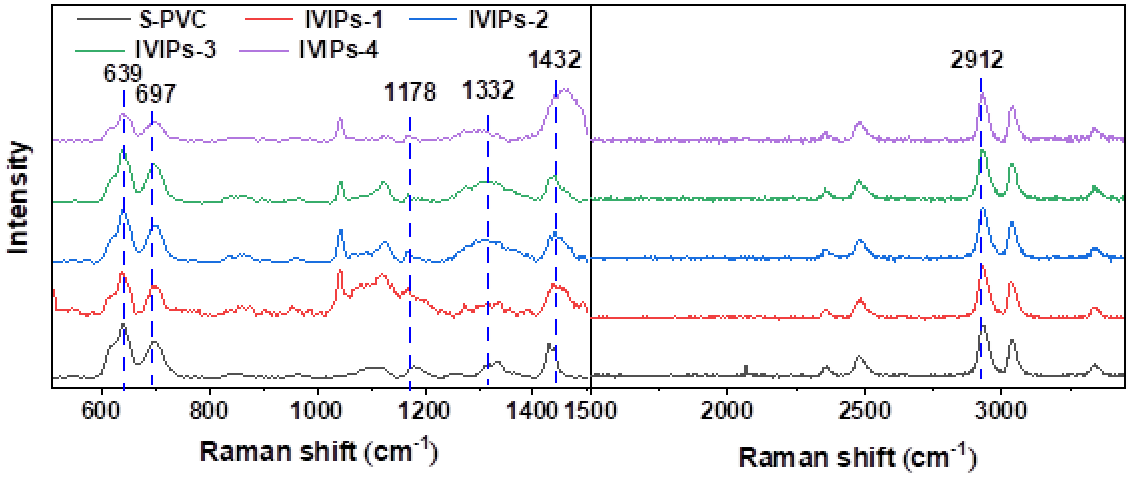


**Figure S3.** Raman spectroscopy of standard PVC and original PVC intravenous infusion products (PVC-IVIPs). Related to Figure 1.

Note: "IVIPs-i" represents different part of PVC-IVIPs (i=1, 2, 3, 4).

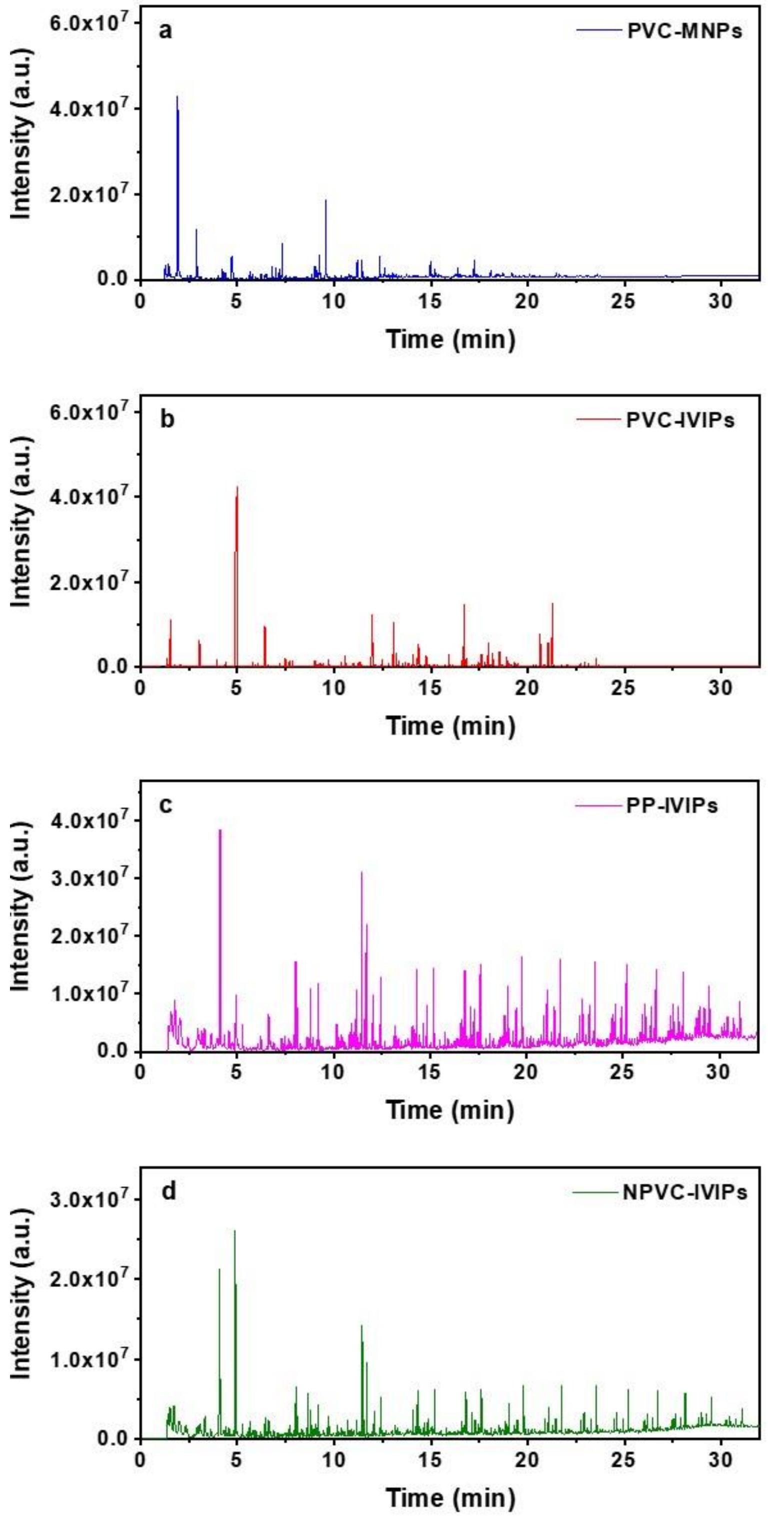


**Figure S4.** Total ion chromatogram (TIC) of standard polyvinyl chloride (PVC) micro(nano)plastics (MNPs) and intravenous injection products (IVIPs) composed of different materials. Related to Figure 1.

Note: **a**, standard PVC micro(nano)plastics (PVC-MNPs); **b**, PVC based intravenous infusion products (PVC-IVIPs); **c**, polypropylene (PP) based intravenous infusion products (PP-IVIPs); **d**, non-PVC multilayer co-extrusion film based intravenous infusion products (NPVC-IVIPs).

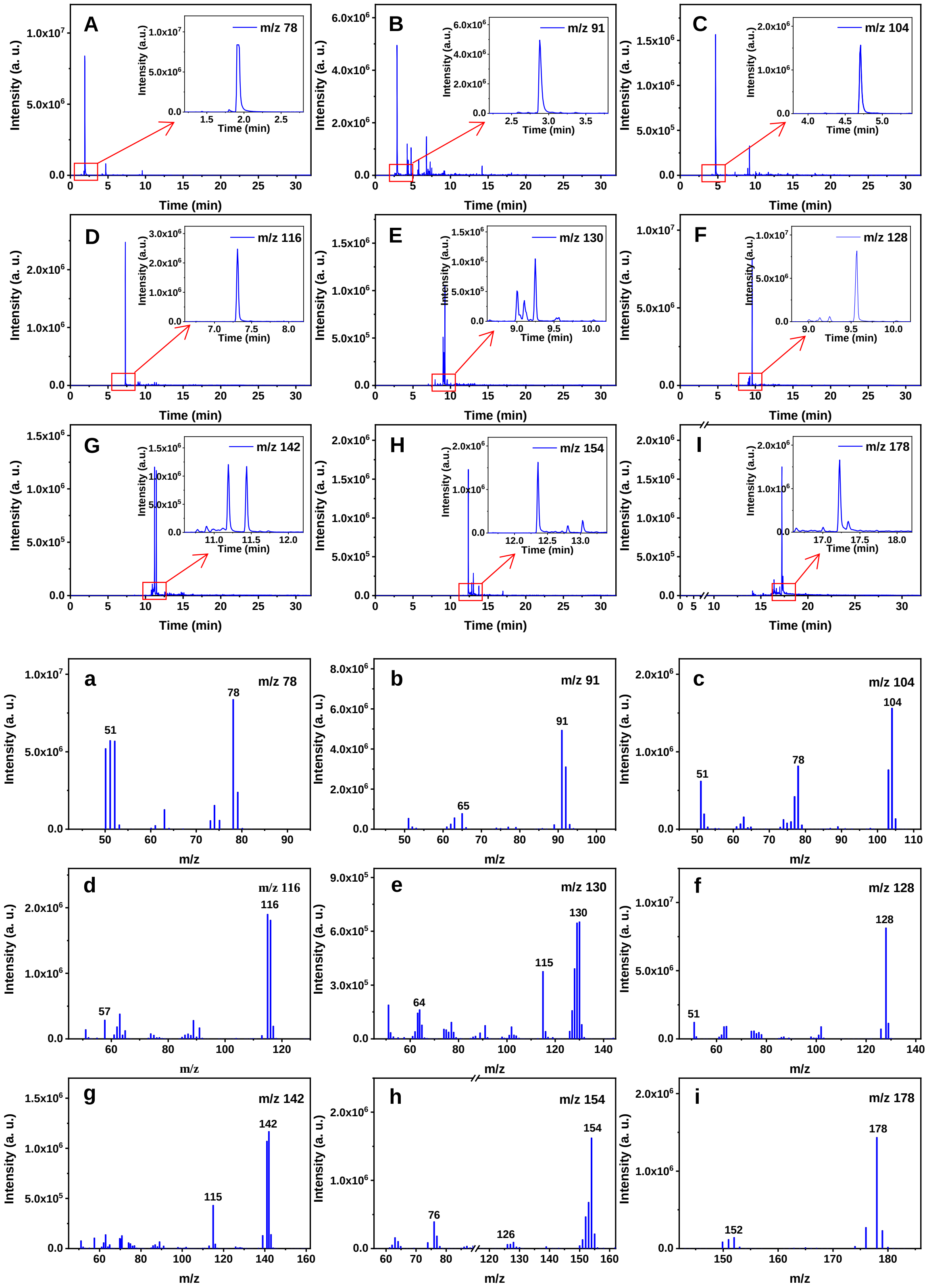


**Figure S5.** Chromatograms of standard PVC-MNPs. Related to Figure 1.

Note: **A~I**, selected indicator-ion chromatograms (SIC); **a~i**, mass spectrum of corresponding indicator ion. Each arrow indicates a magnified view of the area inside the red square.

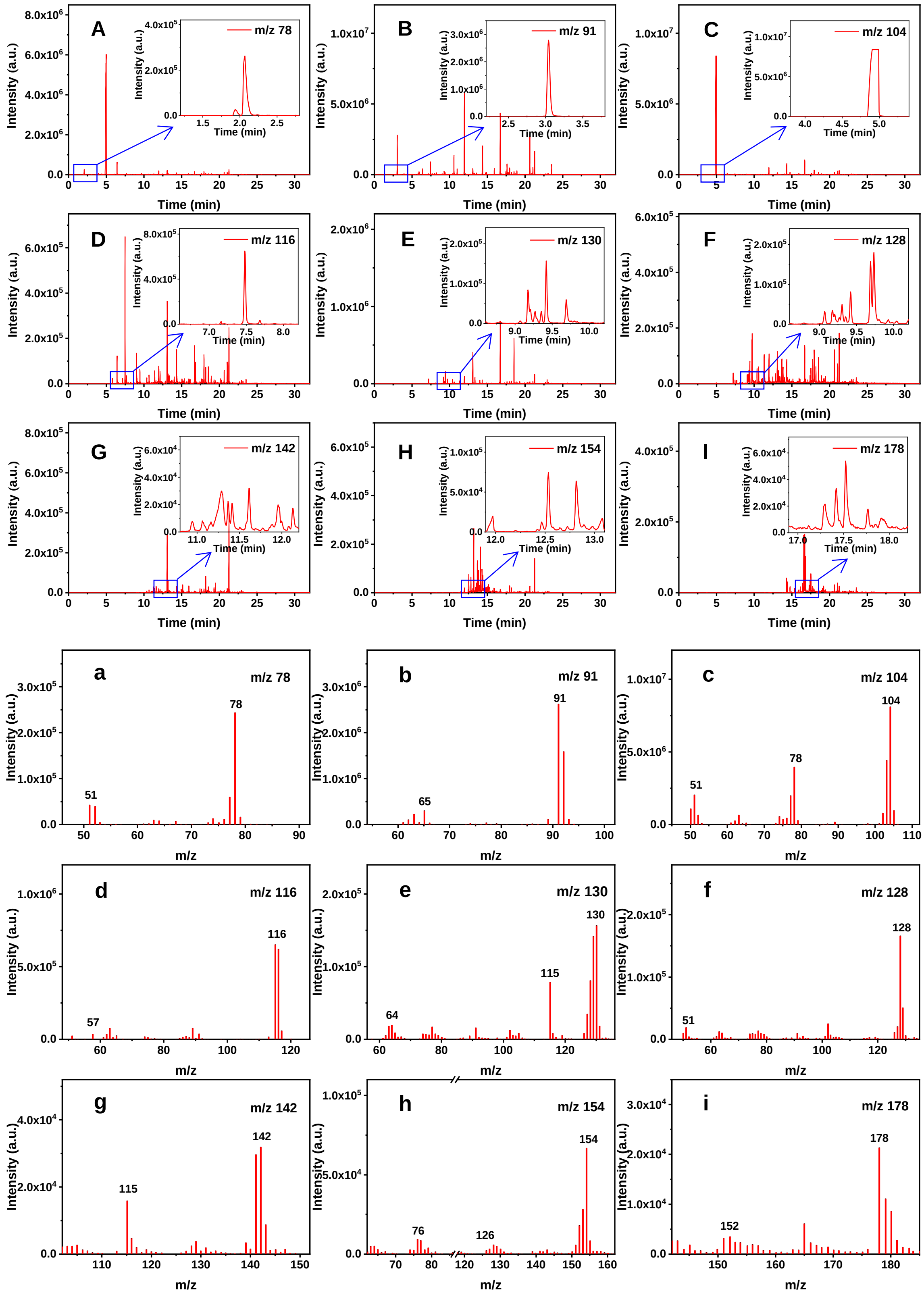


**Figure S6.** Chromatograms of original PVC based intravenous infusion products. Related to Figure 1.

Note: **A~I**, selected indicator-ion chromatograms (SIC); **a~i**, mass spectrum of corresponding indicator ion. Each arrow indicates a magnified view of the area inside the red square.

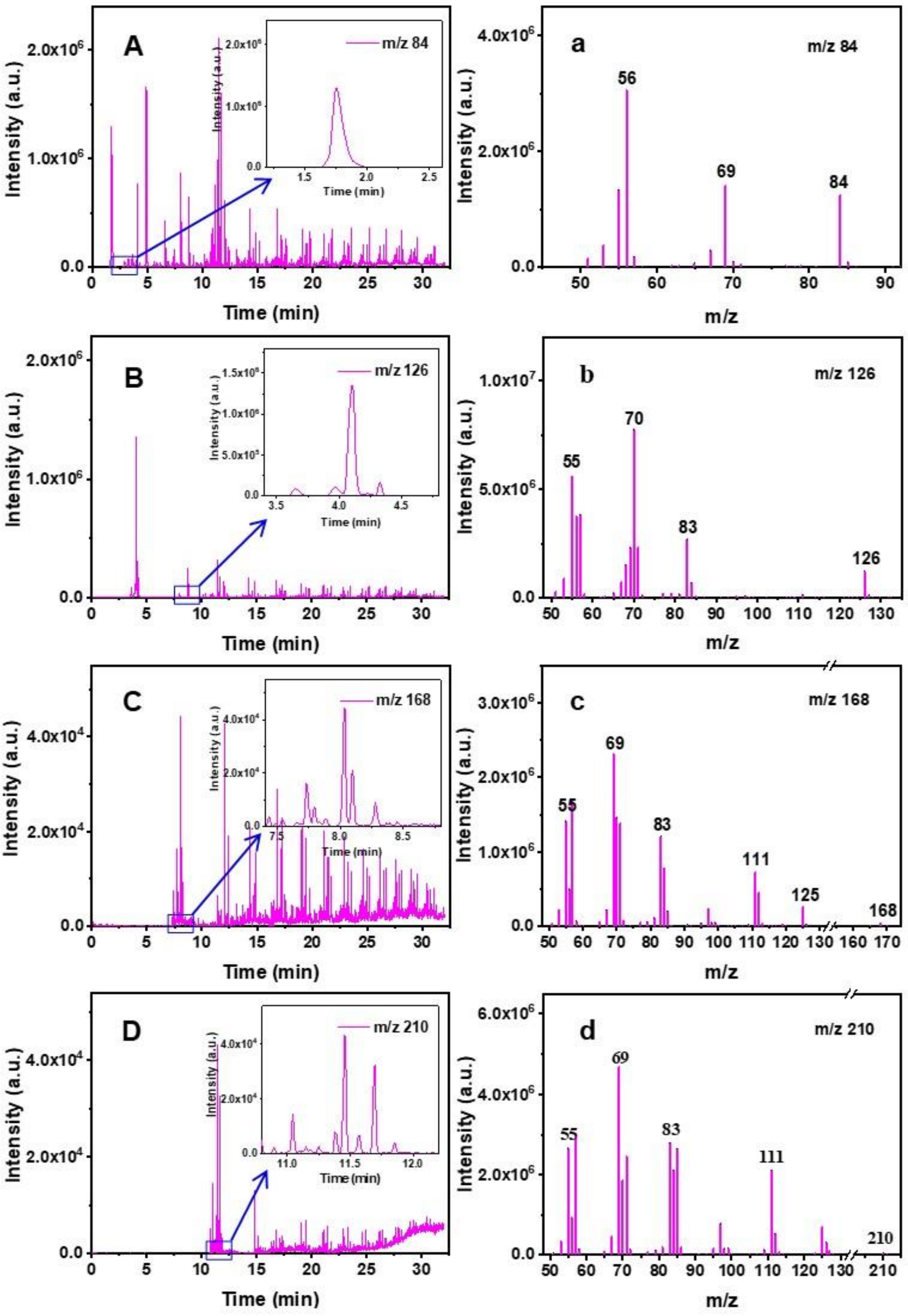


**Figure S7.** Chromatograms of original polypropylene (PP) based intravenous infusion products. Related to Figure 1.

Note: **A~D**, selected indicator-ion chromatograms (SIC); **a~d**, mass spectrum of corresponding indicator ion. Each arrow indicates a magnified view of the area inside the blue square.

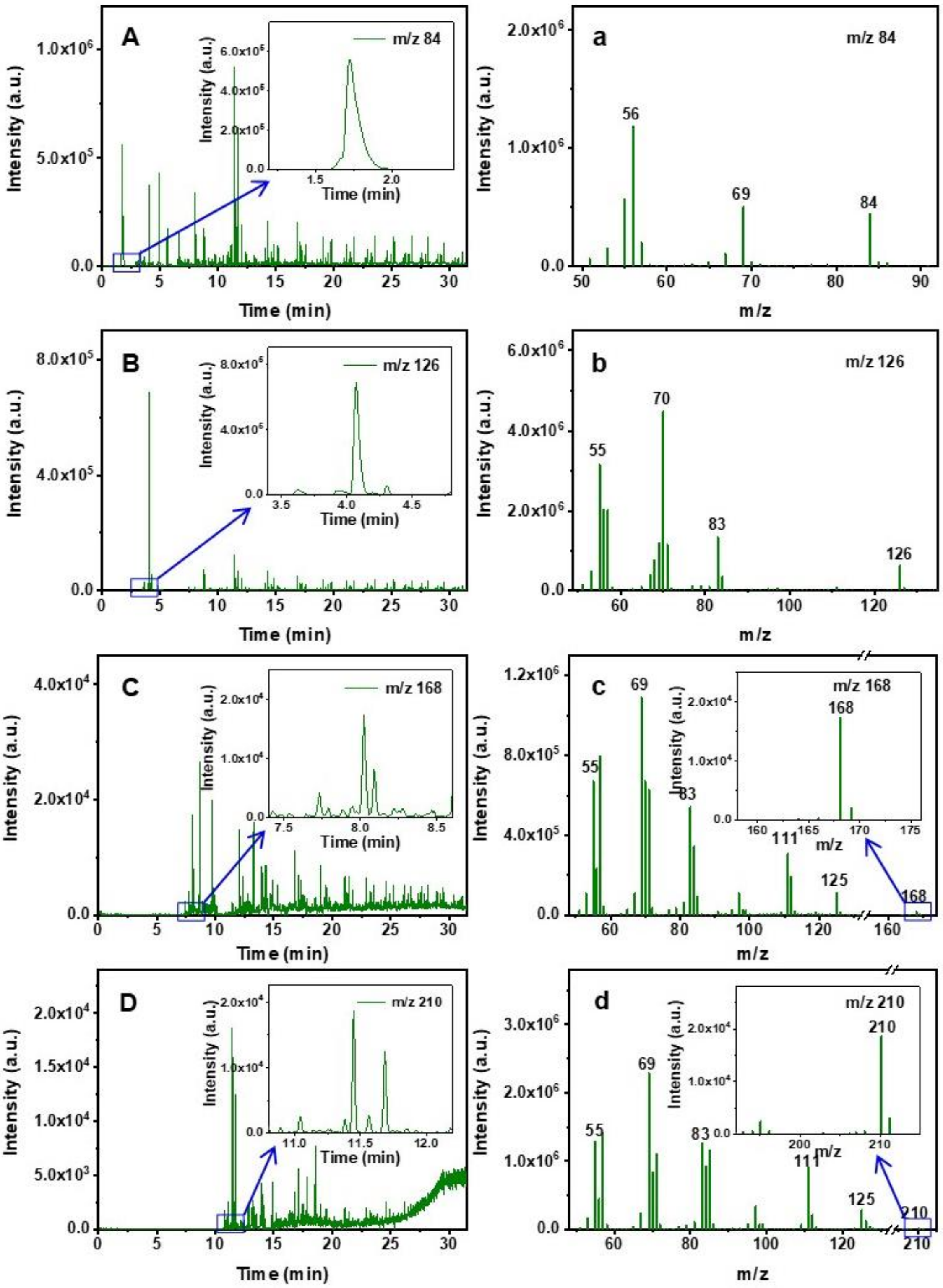


**Figure S8.** Chromatograms of original non-PVC multilayer co-extrusion film (NPVC) based intravenous infusion products. Related to Figure 1.

Note: **A~D**, selected indicator-ion chromatograms (SIC); **a~d**, mass spectrum of corresponding indicator ion. Each arrow indicates a magnified view of the area inside the blue square.

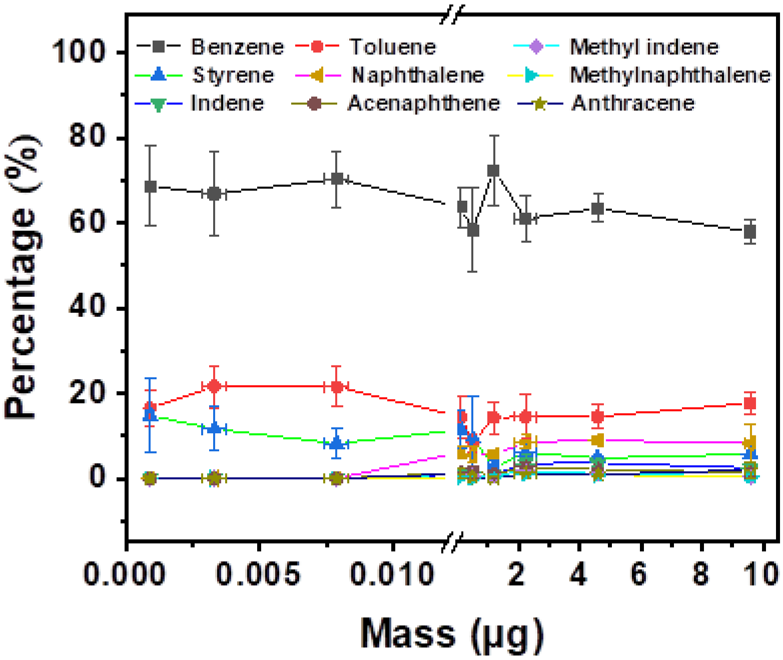


**Figure S9.** Relationship between the mass of standard PVC and the percentages of their pyrolysis products based on Py-GC-MS measurements. Related to Figure 1.

Note: The percentages of pyrolysis products was calculated by $P_i = \frac{A_i}{A_1+A_2+\cdots+A_9}$, where $P_i$ represents the percentage of certain pyrolysis product; $A_i$ represents the peak areas of pyrolysis product $i$ ($i$= 1, 2,..., 9); $A_1$, $A_2$,...$A_9$, represent the peak area of benzene (m/z 78), methylbenzene (m/z 91), styrene (m/z 104), indene (m/z 116), methyl indene(m/z 130), naphthalene(m/z 128), methylnaphthalene(m/z 142), acenaphthene (m/z 154) and anthracene (m/z 178), respectively.

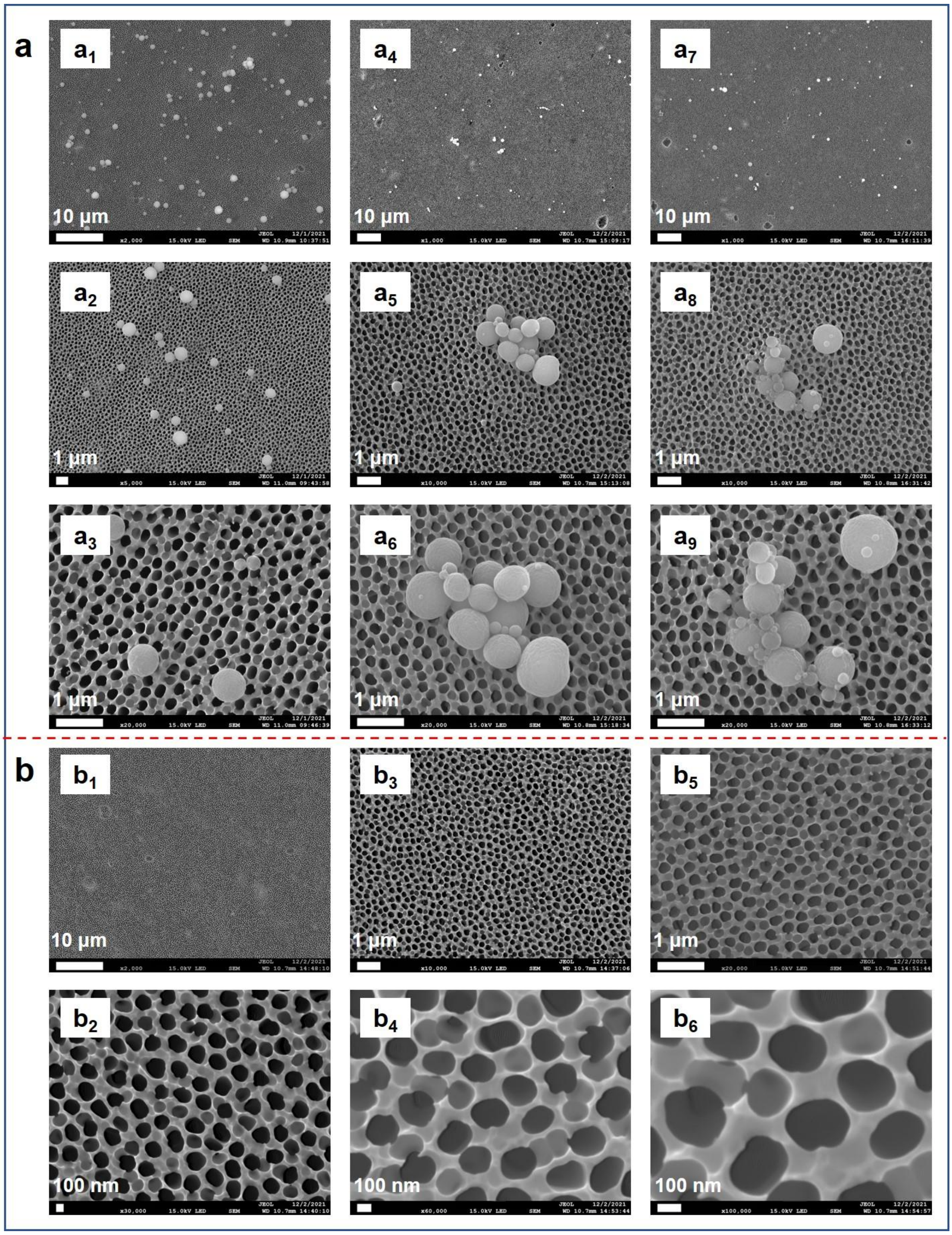


**Figure S10.** Typical SEM images of spiked PVC and $Al_2O_3$ membranes. Related to Figure 3.

Note: **a**, typical SEM images of spiked PVC collected on $Al_2O_3$ membranes. **$a_1$~$a_3$**, spiked PVC (5 μg) was collected by direct dropping on $Al_2O_3$ membrane, **$a_4$~$a_6$**, PVC was spiked in NS and packed in polypropylene (PP) bottles, **$a_7$~$a_9$**, PVC was spiked GI and packed by PP bottles. **b**, procedure blanks, $Al_2O_3$ membranes after filtering 0.9% sodium chloride injection (normal saline, NS) or 5% glucose injection (GI) packed in PP bottles (500 mL) without spiking of PVC.

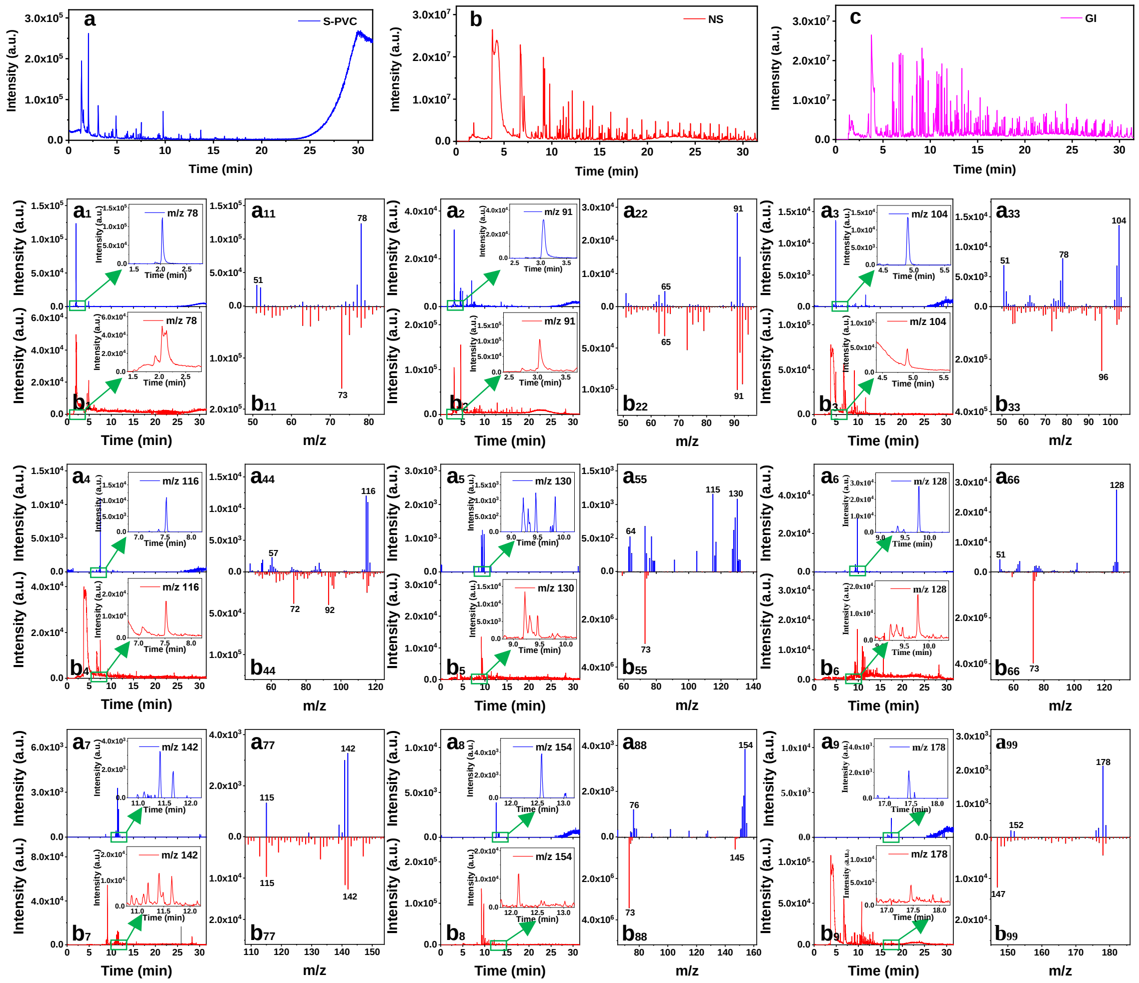


**Figure S11.** Compared chromatogram of standard PVC with procedure blank determined by Py-GC-MS. Related to Figure 2.

Note: **a**: Total ion chromatogram (TIC) of standard PVC; **b**: Total ion chromatogram (TIC) of procedure blank, which is prepared by filtering NS (3000 mL); **c**: Total ion chromatogram (TIC) of procedure blank, which is prepared by filtering GI (3000 mL). **Blue**: selected indicator-ion chromatograms (SIC) and mass spectrum (MS) of standard PVC; **Red**: SIC and MS of procedure blank, which is filtered NS; **$a_1$~$a_9$** represent selected indicator-ion chromatograms (SIC) of benzene, methylbenzene, styrene, indene, methyl indene, naphthalene, methylnaphthalene, acenaphthene and anthracene, respectively; **$b_1$~$b_9$** represent SIC of benzene, methylbenzene, styrene, indene, methyl indene, naphthalene, methylnaphthalene, acenaphthene and anthracene, respectively; **$a_{11}$~$a_{99}$** represent mass spectrum (MS) of benzene, methylbenzene, styrene, indene, methyl indene, naphthalene, methylnaphthalene, acenaphthene and anthracene, respectively; **$b_{11}$~$b_{99}$** represent MS of benzene, methylbenzene, styrene, indene, methyl indene, naphthalene, methylnaphthalene, acenaphthene and anthracene, respectively. Each arrow indicates a magnified view of the area inside the green square.

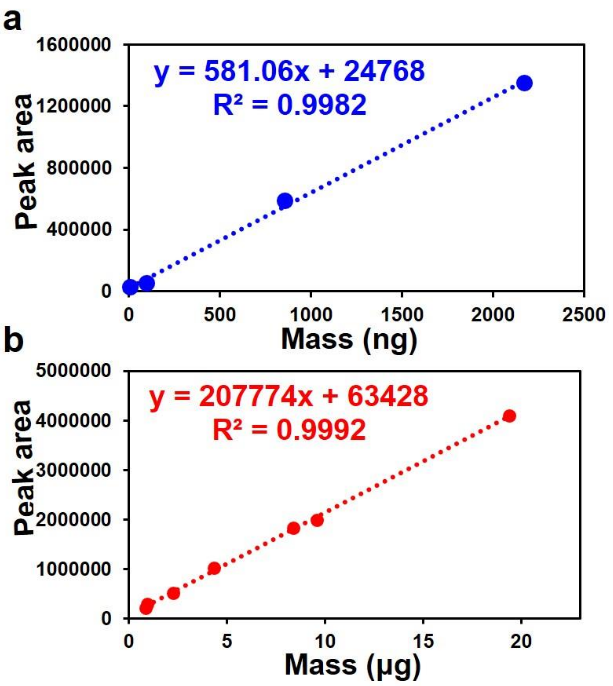


**Figure S12.** Calibration curves prepared by plotting peak area of benzene (m/z 78) against the quantities of standard PVC. Related to Figure 5.

Note: Benzene (m/z 78) is the indicator ion of PVC in Py-GC-MS determination. **a,** the mass of PVC ranged from 8.27 to 2307 ng; **b,** the mass of PVC ranged from 0.87 to 19.37 μg.

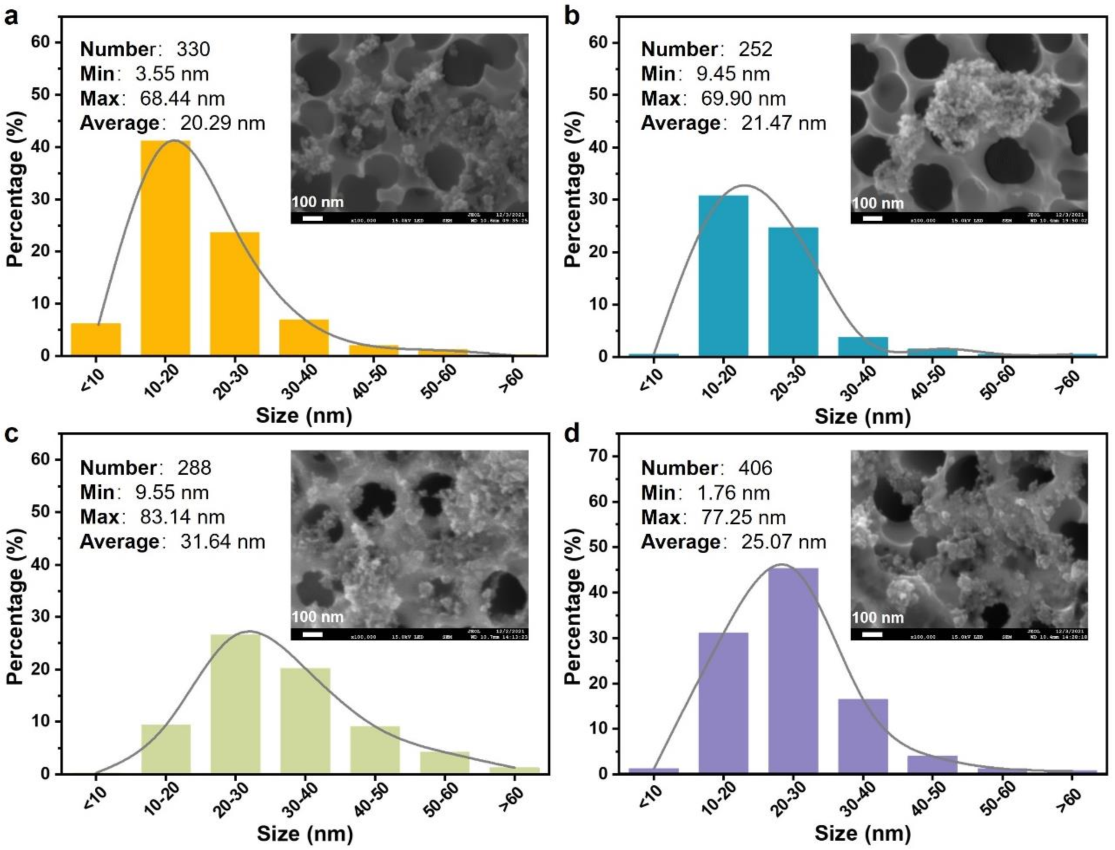


**Figure S13.** Typical SEM images and size distributions of released MNPs from PVC-IVIPs. Related to Figure 3.

Note: **a**, collected PVC-MNPs by filtering 0.9% sodium chloride injection (normal saline, NS) packed in polyvinyl chloride (PVC) bags. **b**, collected PVC-MNPs by filtering NS packed in non-PVC multilayer co-extrusion film infusion bag (NPVC). **c**, collected PVC-MNPs by filtering 5% glucose injection (GI) packed in PVC bags. **d**, collected PVC-MNPs by filtering GI packed in NPVC.

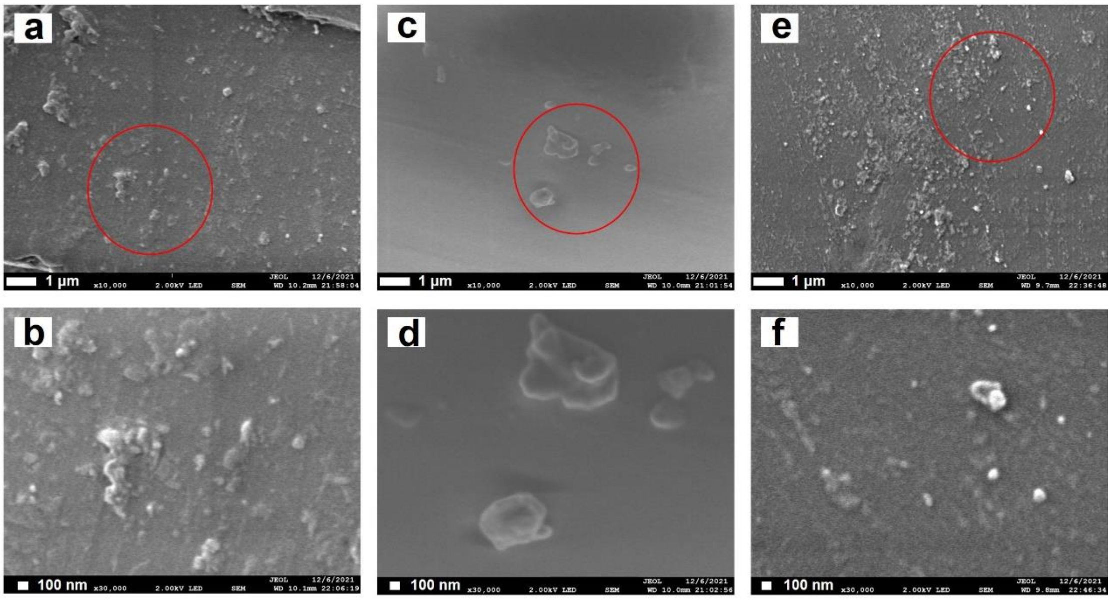


**Figure S14.** Typical SEM images of original PVC intravenous infusion products. Related to Figure 3.

Note: **a** and **b**, PVC bags; **c~f**, PVC tubes. **b, d,** and **f** (scale bar 1 μm) are the magnified view of the area inside the red square of **a, c,** and **e** (scale bar 100 nm), respectively.

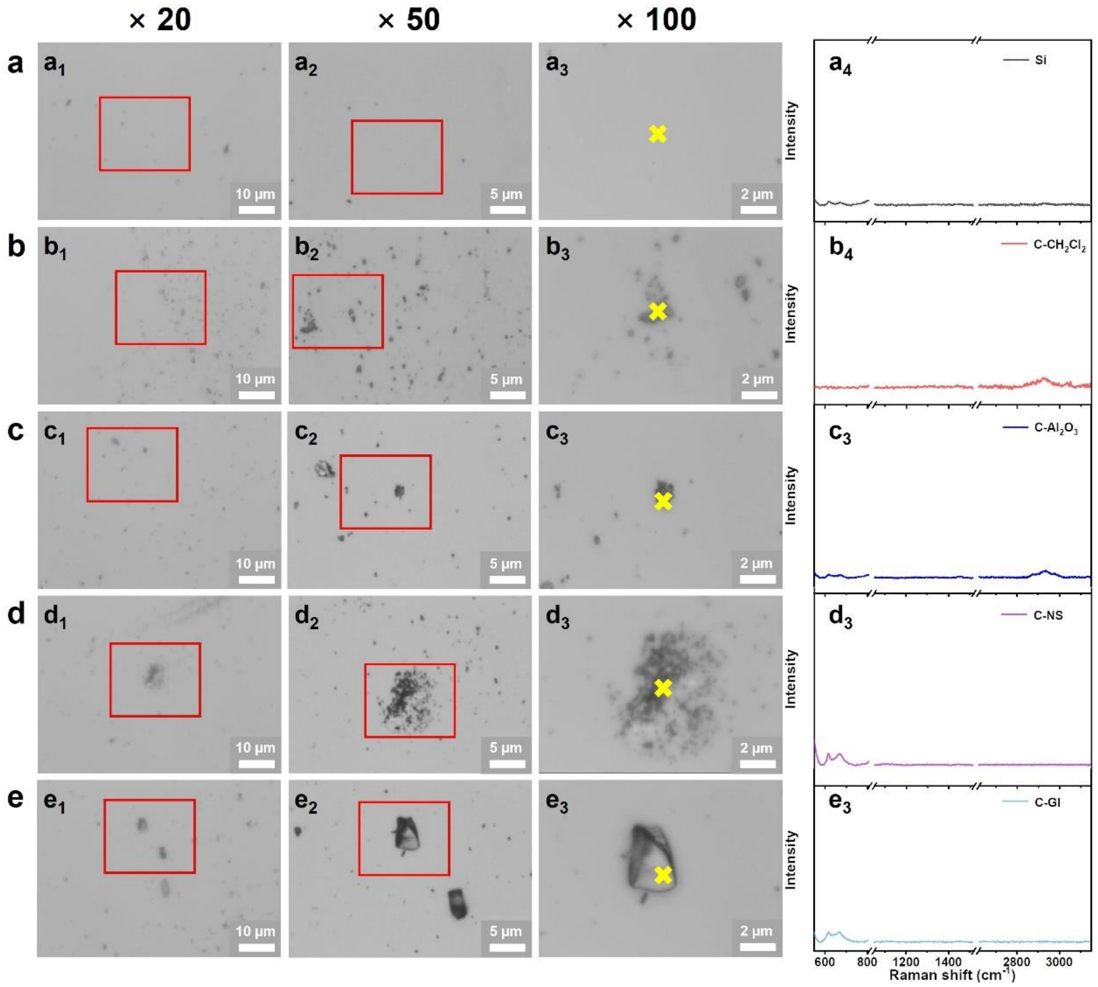


**Figure S15.** Optical images and Raman spectra of strict quality control. Related to Figure 4.

Note: **a**, pristine silicon wafer; **b**, dichloromethane (DCM) without any plastics directly dropped on silicon wafer; **c**, pristine $Al_2O_3$ membrane without any filtration treated by DCM; **d**, directly filtering 500 mL of 0.9% sodium chloride injection (normal saline, NS) packed in non-PVC multilayer co-extrusion film infusion bag (NPVC) bags onto $Al_2O_3$ membrane (C-NS); **e**, filtering 500 mL of 5% glucose injection (GI) packed in NPVC bags onto $Al_2O_3$ membrane (C-GI). Optical images of **$a_1$-$e_1$**, **$a_2$-$e_2$** and **$a_3$-$e_3$** were obtained under an objective lens of 20×, 50× and 100×, respectively. **$a_2$-$e_2$** represent a magnified view of red box in **$a_1$-$e_1$**, respectively. **$a_3$-$e_3$** represent a magnified view of red box in **$a_2$-$e_2$**, respectively. Raman spectra of **$a_4$-$e_4$** were obtained at the marked positions in **$a_3$-$e_3$**, respectively.

**Supplementary References**


1. Sahnoune, M., Tokhadzé, N., Devémy, J., et al. (2021). Understanding and characterizing the drug sorption to PVC and PE materials. ACS Appl. Mater. Inter. *13*, 18594-18603. https://doi.org/10.1021/acsami.1c03284
2. Sparrow, N. Data from "Medical-Grade PVC just what the doctor (and recycler) ordered". Available at https://www.plasticstoday.com/medical/medical-grade-pvc-just-what-doctor-and-recycler-ordered. Deposited 15 September 2021.
3. Matsui, K., Ishimura, T., Mattonai, M., et al. (2020). Identification algorithm for polymer mixtures based on Py-GC/MS and its application for microplastic analysis in environmental samples. J. Anal. Appl. Pyrol. *149*, 104834. https://doi.org/10.1016/j.jaap.2020.104834
4. Blancho, F., Davranche, M., Hadri, H. E., et al. (2021). Nanoplastics identification in complex environmental matrices: Strategies for polystyrene and polypropylene. Environ. Sci. Technol. *55*, 8753-8759. https://doi.org/10.1021/acs.est.1c01351
5. Matsueda, M., Mattonai, M., Iwai, I., et al. (2021). Preparation and test of a reference mixture of eleven polymers with deactivated inorganic diluent for microplastics analysis by pyrolysis-GC-MS. J. Anal. Appl. Pyrol. *154*, 104993. https://doi.org/10.1016/j.jaap.2020.104993
6. Ter Halle, A., Jeanneau, L., Martignac, M., et al. (2017). Nanoplastic in the North Atlantic subtropical gyre. Environ. Sci. Technol. *51*, 13689-13697. https://doi.org/10.1021/acs.est.7b03667
7. Coralli, I., Giorgi, V., Vassura, I., et al. (2022) Secondary reactions in the analysis of microplastics by analytical pyrolysis. J. Anal. Appl. Pyrol. *161*, 105377. https://doi.org/10.1016/j.jaap.2021.105377
8. Ribeiro, F., Okoffo, E. D., O'Brien, J. W., et al. (2020). Quantitative analysis of selected plastics in high-commercial-value Australian seafood by pyrolysis gas chromatography mass spectrometry. Environ. Sci. Technol. *54*, 9408-9417. https://doi.org/10.1021/acs.est.0c02337
9. Fischer, M., Scholz-Böttcher, B. M. (2017). Simultaneous trace identification and quantification of common types of microplastics in environmental samples by pyrolysis-gas chromatography-mass spectrometry. Environ. Sci. Technol. *51*, 5052-5060. https://doi.org/10.1021/acs.est.6b06362
10. da Costa, J. P., Reis, V., Paço, A., et al. (2019). Micro (nano) plastics–analytical challenges

towards risk evaluation. TrAC Trends Anal. Chem. *111*, 173-184. https://doi.org/10.1016/j.trac.2018.12.013

11. Ivleva, N. P. (2021). Chemical analysis of microplastics and nanoplastics: challenges, advanced methods, and perspectives. Chem. Rev. *121*, 11886-11936. https://doi.org/10.1021/acs.chemrev.1c00178
12. Hartmann, N. B. Huffer, T., Thompson, R. C., et al. (2019). Are we speaking the same language? Recommendations for a definition and categorization framework for plastic debris. Environ. Sci. Technol. *53*, 1039-1047. https://doi.org/10.1021/acs.est.8b05297
13. Infusion equipment for medical use part 4: infusion sets for single use, gravity feed. BS EN ISO 8536-4: 2013+A1: (2013).
14. Xu, Y., Ou, Q., Jiao, M., et al. (2022). Identification and quantification of nanoplastics in surface water and groundwater by pyrolysis gas chromatography–mass spectrometry. Environ. Sci. Technol. *56*, 4988-4997. https://doi.org/10.1021/acs.est.1c07377
15. Lai, Y., Dong, L., Li, Q., et al. (2021). Counting nanoplastics in environmental waters by single particle inductively coupled plasma mass spectroscopy after cloud-point extraction and in situ labeling of gold nanoparticles. Environ. Sci. Technol. *55*, 4783-4791. https://doi.org/10.1021/acs.est.0c06839
